# Targeting legumain-mediated cell-cell interaction sensitizes glioblastoma to immunotherapy in preclinical models

**DOI:** 10.1172/JCI186034

**Published:** 2025-03-25

**Authors:** Lizhi Pang, Songlin Guo, Yuyun Huang, Fatima Khan, Yang Liu, Fei Zhou, Justin D. Lathia, Peiwen Chen

**Affiliations:** 1 Department of Cancer Biology, Lerner Research Institute, Cleveland Clinic, Cleveland, Ohio, USA.; 2 Department of Neurological Surgery, Feinberg School of Medicine, Northwestern University, Chicago, Illinois, USA.; 3 Department of Cardiovascular and Metabolic Sciences, Lerner Research Institute, Cleveland Clinic, Cleveland, Ohio, USA.; 4 Cleveland Clinic Lerner College of Medicine of Case Western Reserve University, Cleveland, Ohio, USA.; 5 Rose Ella Burkhardt Brain Tumor & Neuro-Oncology Center, Cleveland Clinic, Cleveland, Ohio, USA.; 6 Case Comprehensive Cancer Center, Cleveland, Ohio, USA.

**Keywords:** Immunology, Oncology, Brain cancer, Immunotherapy, Macrophages

## Abstract

Tumor-associated macrophages (TAMs) are the most prominent immune cell population in the glioblastoma (GBM) tumor microenvironment and play critical roles in promoting tumor progression and immunosuppression. Here we identified that TAM-derived legumain (LGMN) exhibited a dual role in regulating the biology of TAMs and GBM cells. LGMN promoted macrophage infiltration in a cell-autonomous manner by activating the GSK3β/STAT3 pathway. Moreover, TAM-derived LGMN activated integrin α_v_/AKT/p65 signaling to drive GBM cell proliferation and survival. Targeting of LGMN-directed macrophage (inhibiting GSK3β and STAT3) and GBM cell (inhibiting integrin α_v_) mechanisms resulted in an antitumor effect in immunocompetent GBM mouse models that was further enhanced by combination with anti–PD-1 therapy. Our study reveals a paracrine and autocrine mechanism of TAM-derived LGMN that promotes GBM progression and immunosuppression, providing effective therapeutic targets to improve immunotherapy in GBM.

## Abstract 2

The study reveals a paracrine and autocrine mechanism of TAM-derived LGMN in promoting GBM progression and provides effective therapeutic targets for improving immunotherapy in GBM.

## Graphical abstract

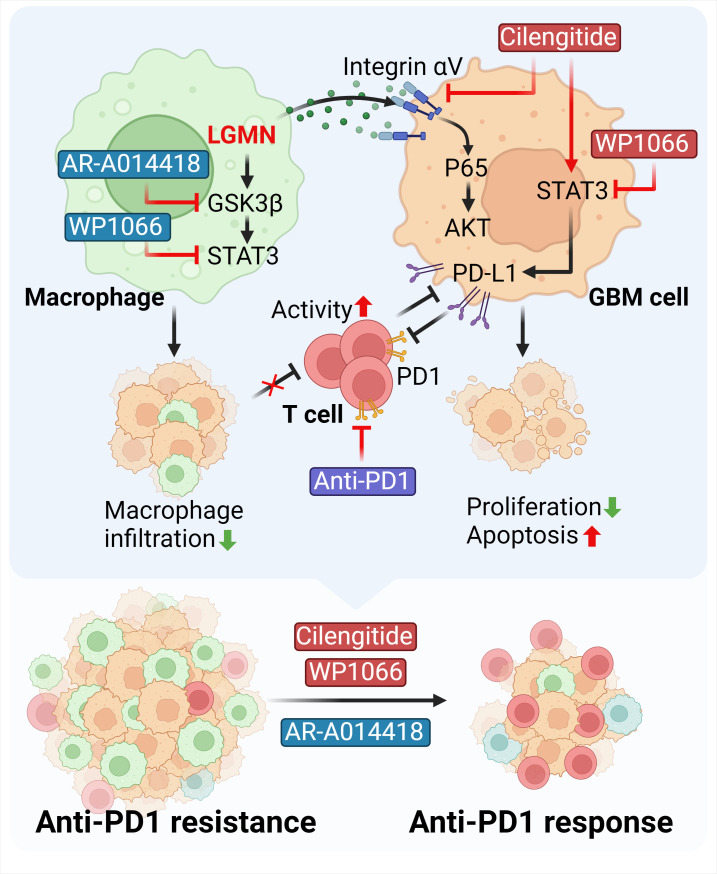

## Introduction

Glioblastoma (GBM) is the most common primary malignant brain tumor in adults (1, 2). The current standard of care, including maximal surgical resection followed by chemoradiotherapy, only modestly extends the survival of GBM patients (3). Despite these aggressive treatments, the 5-year survival rate is still less than 10% (1, 2). There is an urgent need to develop effective treatments to combat this fatal disease. Since immunotherapies show long-term remissions in many other cancers (4, 5), early studies were prompted to test the effectiveness of immunotherapies for GBM (6–9). However, the data of several clinical trials on immune checkpoint inhibitors (ICIs) did not show a meaningful benefit for GBM patients (9–13). The unsatisfying clinical trial results are in part due to the immunosuppressive GBM tumor microenvironment (TME) (14–16), which is composed of various immune cell populations, such as tumor-associated macrophages (TAMs), myeloid-derived suppressor cells, and neutrophils (17, 18). As the most prominent population of immune cells in GBM tumor tissues, TAMs constitute up to 50% of total cells, by far outnumbering T cells in tumor tissues (15, 19). Thus, GBM is considered a typical immunologically “cold” tumor that barely responds to single-agent ICI therapy (17, 20, 21). Understanding the molecular basis of TAM biology is essential for enhancing immunotherapy efficiency in GBM patients.

We recently demonstrated that *PTEN*-null GBM cells could secrete lysyl oxidase (LOX) to the TME, resulting in TAM infiltration by activating integrin β_1_ (22). Aside from the LOX/integrin β_1_ axis, recent studies have identified other chemokine-receptor pairs, such as OPN–integrin α_v_β_5_, CSF1-CSF1R, TFPI2–integrin α_v_, SLIT2-ROBO1/2, and CCL2/CCL7-CCR2, that are critical for TAM infiltration (23–27). These findings support the idea that chemokine-receptor pairs between GBM cells and TAMs can be targeted to regulate TAM biology and antitumor immunity in GBM (14, 15, 20, 28). Recent studies using single-cell technologies have revealed that TAMs are a heterogeneous and plastic population of cells in GBM (17, 29–32). Therefore, TAM function and infiltration may not be determined by a single chemokine-receptor pair, and targeting such single chemokine-receptor pairs may not generate significant antitumor effect. For instance, treatment with PLX3397 (a CSF1R inhibitor) failed to extend survival of recurrent GBM patients in a phase II clinical trial (33). These findings suggest that consideration of the framework of context-dependent interactions should be incorporated into the development of therapeutic approaches for targeting the GBM-TAM symbiosis (14, 15, 18, 20, 23). One such context example is hypoxia, a key GBM hallmark, that significantly influences TAM biology (34–36). Our recent studies have demonstrated that hypoxia-triggered legumain (LGMN) is highly enriched in TAMs and required for promoting macrophage immunosuppressive polarization (37, 38).

LGMN is a member of the C13 family of peptidases that cleaves peptide bonds on the C-terminal side of asparagine residues (39). LGMN is highly expressed in different types of tumors and correlated with poor prognosis (40). In GBM, consistent with our previous observation (37), a recent study revealed that LGMN could sustain GBM tumor growth under hypoxic conditions (41). However, it remains to be determined whether and how TAM-derived LGMN regulates macrophage infiltration and GBM cell biology. Here, we used single-cell RNA-Seq (scRNA-Seq) analysis followed by functional studies to show that TAM-derived LGMN promotes tumor progression and immunosuppression by dually targeting GBM cells and macrophages through a mechanism of activating the glycogen synthase kinase 3β (GSK3β)/signal transducer and activator of transcription 3 (STAT3) and integrin α_v_/protein kinase B (AKT)/NF-κB p65 (p65) pathways, respectively. Cotargeting the LGMN-directed cell-cell interaction inhibits tumor progression and overcomes the resistance to anti–PD-1 therapy in GBM mouse models. Collectively, this study provides evidence to support cotargeting LGMN downstream signals in GBM cells and macrophages to inhibit GBM progression and overcome immunotherapy resistance.

## Results

### LGMN promotes macrophage infiltration in GBM.

**Figure 1:**
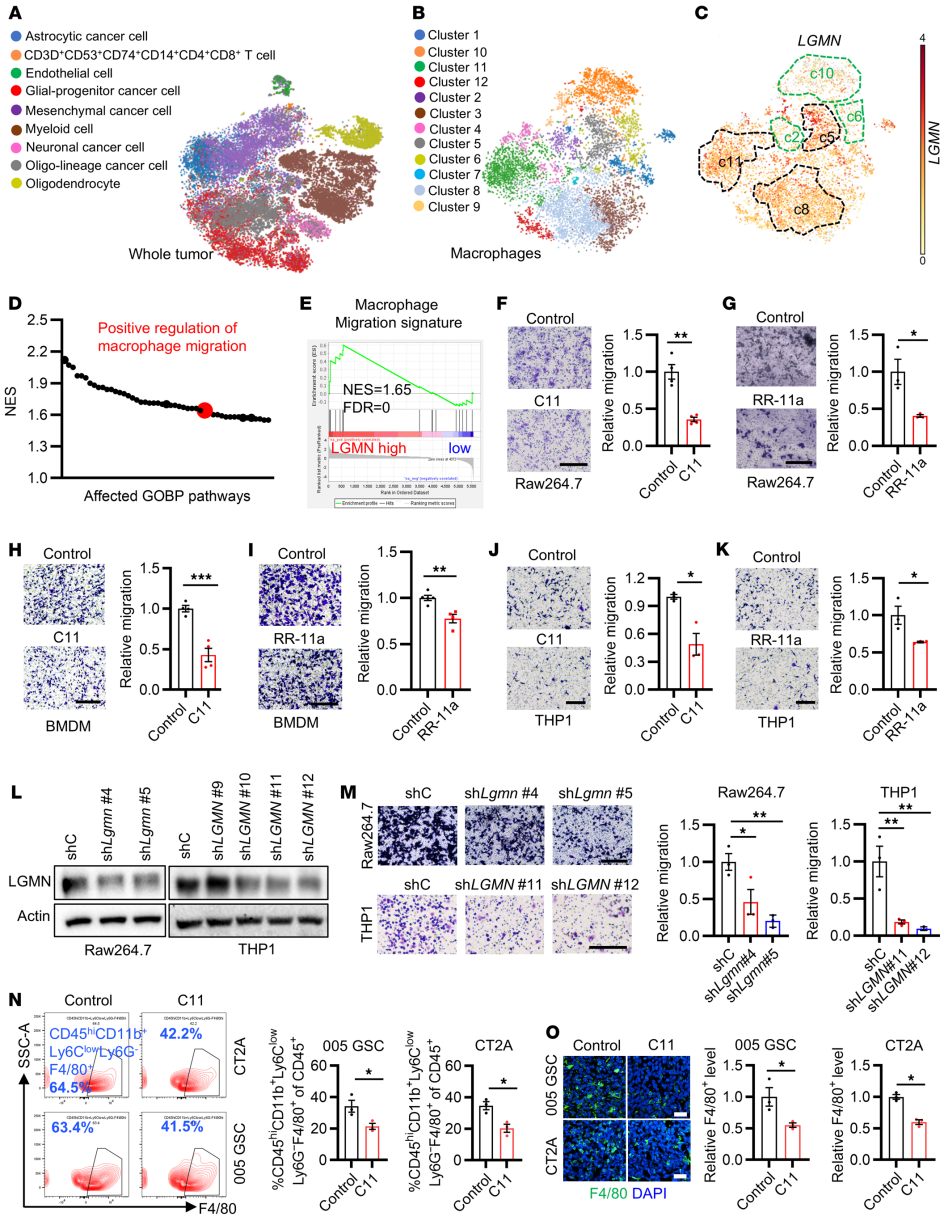
LGMN promotes macrophage infiltration in GBM. (**A** and **B**) High-resolution uniform manifold approximation and projection (UMAP) plots of 9 types of cells (**A**) and 12 subclusters of macrophages (**B**) in GBM patient tumors. The analysis was based on the scRNA-Seq dataset (EGAS00001004422). (**C**) UMAP showing expression of *LGMN* in macrophage subpopulations. Darker color represents higher *LGMN* expression. (**D**) GSEA on Gene Ontology biological process (GOBP) signatures showing enriched pathways in the *LGMN-*high macrophage group. NES, normalized enrichment score. (**E**) GSEA showing enrichment of macrophage migration signature in macrophages with high compared with low *LGMN*. NES and FDR *q* values are shown. (**F** and **G**) Representative images and quantification of relative migration of Raw264.7 macrophages after treatment with C11 (1 μmol/L; **F**) and RR-11a (20 nmol/L; **G**). *n* = 4 independent samples. (**H** and **I**) Representative images and quantification of relative migration of BMDMs after treatment with C11 (1 μmol/L; **H**) and RR-11a (20 nmol/L; **I**). *n* = 4–5 independent samples. (**J** and **K**) Representative images and quantification of relative migration of THP1 macrophages after treatment with C11 (1 μmol/L; **J**) and RR-11a (20 nmol/L; **K**). *n* = 3 independent samples. (**L**) Immunoblots for LGMN in lysates of Raw264.7 and THP1 macrophages expressing shRNA control (shC) and LGMN shRNAs (sh*LGMN*). (**M**) Representative images and quantification of relative migration of mouse Raw264.7 and human THP1 macrophages expressing shC and sh*LGMN*. *n* = 3 independent samples. (**N**) Representative images and quantification of flow cytometry for percentage of CD45^hi^CD11b^+^Ly6C^lo^Ly6G^–^F4/80^+^ macrophages in size-matched control and C11-treated 005 GSC and CT2A tumors in C57BL/6 mice. C11 (10 mg/kg/d) was administered i.p. in tumor-bearing mice. *n* = 3 independent samples. (**O**) Immunofluorescence and quantification of relative F4/80^+^ macrophages in tumors from the 005 GSC and CT2A GBM mouse models treated with or without C11 (10 mg/kg, i.p., daily). *n* = 3 independent samples. Student’s *t* test (**F**–**K**, **N**, and **O**); 1-way ANOVA test (**M**). *P < 0.05, **P < 0.01, ***P < 0.001. Scale bars: 200 μm (**F–K** and **M**); 25 μm (**O**).

Since LGMN is highly expressed in immunosuppressive TAMs compared with other cell populations in the GBM TME (37), in this study, we hypothesized that LGMN might play an important cell-intrinsic role in macrophages. By analyzing scRNA-Seq data (European Genome-Phenome Archive EGAS00001004422) from newly diagnosed IDH-WT GBM patient tumors (Figure 1A), we identified 12 clusters of tumor-infiltrated macrophage subpopulations (Figure 1B). Among them, macrophage clusters 5, 8, and 11 highly expressed *LGMN*, whereas clusters 2, 6, and 10 showed low *LGMN* expression (Figure 1C and Supplemental Figure 1A; supplemental material available online with this article; https://doi.org/10.1172/JCI186034DS1). These clusters were further grouped as *LGMN-*high TAMs and *LGMN-*low TAMs (Supplemental Figure 1A) for gene set enrichment analysis (GSEA) on the Gene Ontology biological process (GOBP) pathways. Compared with *LGMN-*low TAMs, the *LGMN-*high group correlated with enhanced macrophage migration signature (Figure 1, D and E). Next, we examined The Cancer Genome Atlas (TCGA) GBM dataset using immune cell–related signatures (42–46) and found that high LGMN expression correlated with significantly enriched bone marrow–derived macrophage (BMDM), macrophage, TAM, and monocyte signatures (Supplemental Figure 1, B–E). GOBP analysis on TCGA-GBM data also showed that leukocyte migration, positive regulation of cell motility, and positive regulation of cell migration were the top LGMN-regulated processes (Supplemental Figure 1F).

To confirm the role of LGMN in macrophage migration through experimentation, mouse Raw264.7 macrophages, mouse primary BMDMs, and human THP1 macrophages were seeded into the Transwell insert and treated with or without LGMN inhibitor C11 or RR-11a. The result showed that inhibition of LGMN significantly suppressed macrophage migration (Figure 1, F–K). In addition, we analyzed the trajectories of macrophages by using the Incucyte live imaging system with TrackMate, a single-cell tracking platform (47, 48). The trajectory analysis result demonstrated that C11 and RR-11a treatment significantly reduced the motility of Raw264.7 macrophages, BMDMs, and THP1 macrophages (Supplemental Figure 1, G–L, and Supplemental Videos 1–12). To further confirm the cell-intrinsic effect of LGMN in macrophage migration, we depleted LGMN in Raw264.7 and THP1 macrophages using a shRNA-mediated knockdown system (Figure 1L). Transwell migration assay demonstrated that LGMN depletion significantly inhibited the migration of Raw264.7 and THP1 macrophages (Figure 1M), supporting that LGMN is important for macrophage spontaneous migration. Similarly, Incucyte live cell imaging confirmed that the motility of LGMN-depleted macrophages was significantly slower than that of cells transfected with shRNA control (Supplemental Figure 1M and Supplemental Videos 13–15). Additionally, LGMN recombinant protein significantly increased the migration ability of BMDMs, Raw264.7 macrophages, and U937 macrophages, and this increase was abolished by the treatment with RR-11a or C11 (Supplemental Figure 1, N–P). To investigate whether LGMN is required for chemokine-triggered macrophage migration, Raw264.7 and THP1 macrophages were placed in Transwell inserts with or without the stimulation of a known chemokine, C-C motif chemokine ligand 2 (CCL2), or GBM cell conditioned medium (CM). The results showed that depletion of LGMN in macrophages abolished their migration ability induced by CCL2 or GBM cell CM (Supplemental Figure 1, Q and R). Together, these findings demonstrate that LGMN is required for both spontaneous and directed migration of macrophages.

To investigate the effect of LGMN in macrophage infiltration in vivo, C11, an LGMN inhibitor with a desirable permeability to the blood-brain barrier (49), was used to treat 005 GSC and CT2A tumor–bearing mice. In a flow cytometry assay, CD45^hi^CD11b^+^Ly6C^lo^Ly6G^–^F4/80^+^ (Supplemental Figure 2A) and CD45^hi^CD11b^+^CD68^+^ (Supplemental Figure 2B) were used to define TAMs in both models. We found that C11 treatment significantly decreased TAMs in 005 GSC and CT2A tumors (Figure 1N and Supplemental Figure 2C). Immunofluorescence (IF) analysis also showed that C11-treated tumors had significantly fewer macrophages (F4/80^+^ cells) than control tumors (Figure 1O). To validate whether LGMN affects macrophage survival, we evaluated the proliferation ability of macrophages upon LGMN inhibition. We found that neither LGMN inhibitor (C11 or RR-11a) nor LGMN shRNA knockdown affected the proliferation of Raw264.7 and THP1 macrophages in vitro (Supplemental Figure 2, D–M). Moreover, treatment with the LGMN inhibitor C11 in CT2A and 005 GSC tumor–bearing mice did not affect intratumoral Ki67^+^F4/80^+^ proliferating macrophages (Supplemental Figure 2, N and O). In summary, these findings highlight that the expression of protease LGMN in TAMs promotes macrophage infiltration into the GBM TME.

### TAM-derived LGMN increases macrophage infiltration and tumor progression through the GSK3β/STAT3 axis.

**Figure 2:**
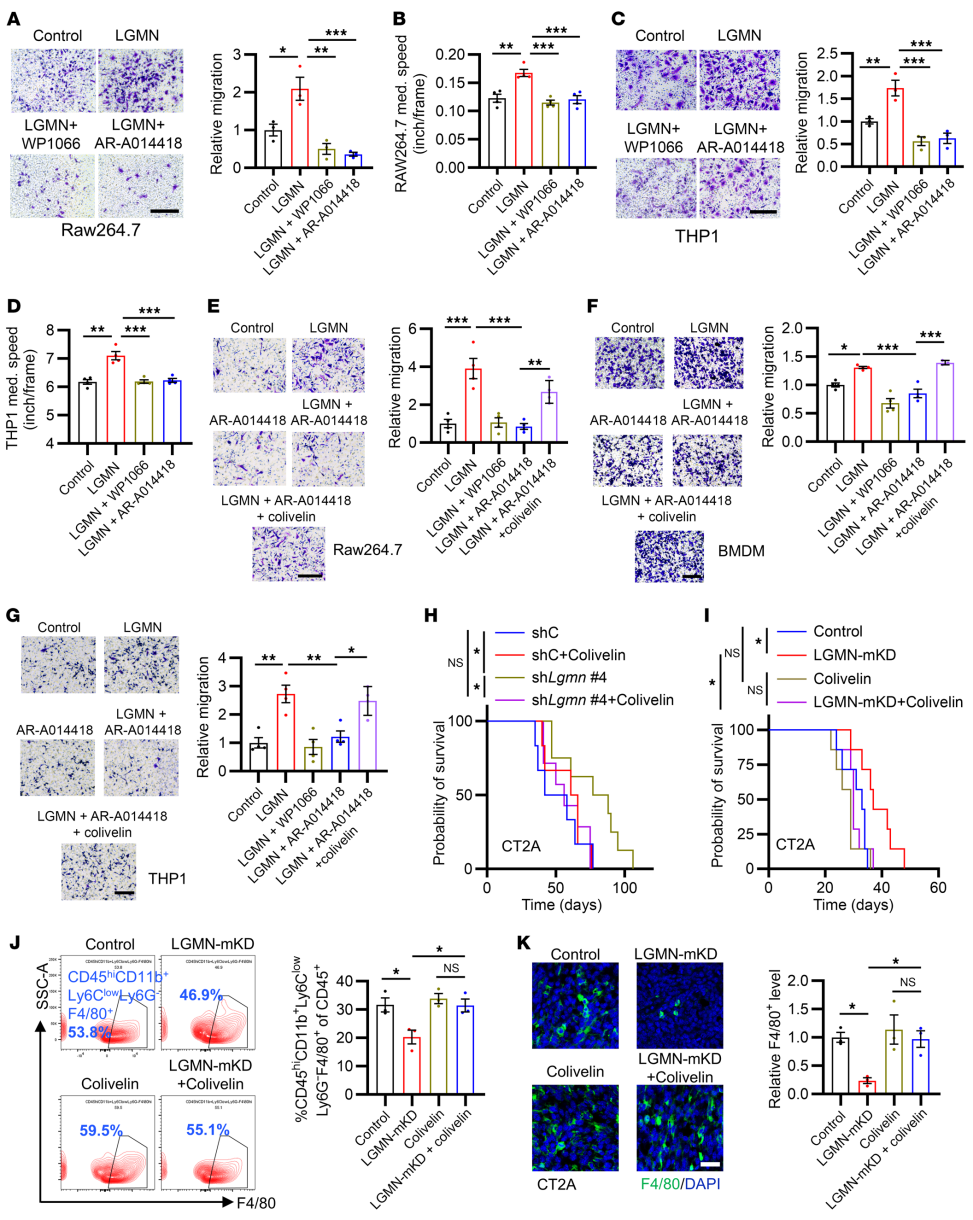
LGMN promotes macrophage migration and tumor progression through the GSK3β/STAT3 axis. (**A**) Relative migration of Raw264.7 macrophages after treatment of LGMN recombinant protein (10 ng/mL) in the presence or absence of STAT3 inhibitor WP1066 (20 nmol/L) or GSK3β inhibitor AR-A014418 (20 nmol/L). (**B**) Quantification of movement speed of Raw264.7 macrophages after treatment of LGMN protein in the presence or absence of WP1066 or AR-A014418 (see Supplemental Videos 16–19). (**C**) Relative migration of THP1 macrophages after treatment of LGMN protein in the presence or absence of WP1066 or AR-A014418. (**D**) Quantification of movement speed of THP1 macrophages after treatment of LGMN protein in the presence or absence of WP1066 or AR-A014418 (see Supplemental Videos 20–23). (**E**–**G**) Relative migration of Raw264.7 macrophages (**E**), BMDMs (**F**), and THP1 macrophages (**G**) pretreated with STAT3 activator Colivelin (30 nmol/L) after treatment of LGMN protein in the presence or absence of AR-A014418. (**H**) Survival curves of C57BL/6 mice implanted with 1 × 10^4^ CT2A cells and 1 × 10^4^ CT2A CM–polarized Raw264.7 cells expressing shC and sh*Lgmn*. Mice were treated with Colivelin (30 mg/kg body weight, i.p., every other day). *n* = 6–8 mice per group. (**I**) Survival curves of CT2A tumor–bearing control and LGMN–macrophage-specific knockdown (LGMN-mKD) mice treated with or without Colivelin. *n* = 6–8 mice per group. (**J**) Flow cytometry for percentage of CD45^hi^CD11b^+^Ly6C^lo^Ly6G^–^F4/80^+^ macrophages in size-matched tumors from control and LGMN-mKD mice treated with or without Colivelin. (**K**) IF and quantification of relative F4/80^+^ macrophages in CT2A tumors from control and LGMN-mKD mice treated with or without Colivelin. *n* = 3 (**A**, **C**, and **J**) or 4 (**B**, **D**, and **G**) independent samples. One-way ANOVA test (**A**–**G**, **J**, and **K**); log-rank test (**H** and **I**). *P < 0.05, **P < 0.01, ***P < 0.001. Scale bars: 200 μm (**A**, **C**, and **G**); 25 μm (**K**).

Our previous study using unbiased human phospho-kinase antibody array has shown that LGMN activates GSK3β and STAT3 in THP1 macrophages (37). Given the crucial role of GSK3β and STAT3 in cell migration (50–53), we hypothesized that activation of GSK3β and STAT3 is essential for LGMN-induced macrophage infiltration in GBM. To test this hypothesis, we pretreated Raw264.7 macrophages with the STAT3 inhibitor WP1066 or the GSK3β inhibitor AR-A014418 before seeding them into a Transwell insert. The Transwell migration assay result showed that either WP1066 or AR-A014418 treatment was sufficient to block LGMN-induced Raw264.7 macrophage migration (Figure 2A). By monitoring the trajectory, we observed that WP1066 and AR-A014418 abolished the acceleration of the movement speed of Raw264.7 macrophages by LGMN (Figure 2B and Supplemental Videos 16–19), indicating that the effect of LGMN on macrophage migration depends on GSK3β and STAT3 signaling. Consistent with results observed in Raw264.7 macrophages, LGMN-induced migration of THP1 human macrophages was negated by the treatment with WP1066 and AR-A014418 (Figure 2, C and D, and Supplemental Videos 20–23). To confirm these findings in vivo, we treated the mice bearing CT2A tumors, where LGMN is highly expressed in TAMs (37), with WP1066 and AR-1014418, and analyzed the change of macrophages in tumor tissues using flow cytometry and IF. The results showed that inhibition of GSK3β or STAT3 significantly reduced intratumoral macrophages (Supplemental Figure 3, A and B).

We have previously shown that GSK3β is upstream of STAT3 in the response to LGMN treatment (37). However, it has yet to be determined whether the GSK3β/STAT3 signaling cascade is responsible for LGMN-induced macrophage migration. To this end, we treated Raw264.7 macrophages, mouse BMDMs, and THP1 macrophages with a potent STAT3 activator, Colivelin (MCE, HY-P1061), in addition to LGMN and AR-A014418. We found that the blocking of LGMN-induced macrophage migration by AR-A014418 was rescued by Colivelin (Figure 2, E–G), suggesting that STAT3 is downstream of GSK3β in the response to LGMN-driven macrophage migration. Since TAMs play a critical role in supporting GBM progression (17, 18, 23, 54, 55), we evaluated whether STAT3 is critical for TAM LGMN–induced GBM growth by co-implanting CT2A cells with immunosuppressive Raw264.7 macrophages harboring shRNA control or *Lgmn* shRNA and treating the tumor-bearing mice with or without Colivelin (Supplemental Figure 3C). The data are consistent with our recent studies showing that knockdown of *Lgmn* in macrophages extended the survival of CT2A tumor–bearing mice (37), and this extension was abolished by the treatment with Colivelin (Figure 2H). Additionally, we generated bone marrow chimeras by transplanting *Lgmn*-knockdown bone marrow cells (Supplemental Figure 3D) into C57BL/6 recipient mice to obtain mice with macrophage-specific knockdown of LGMN (LGMN-mKD mice). After the CT2A tumor implantation, LGMN-mKD mice exhibited significantly lower LGMN expression in TAMs, but not in cancer cells (Supplemental Figure 3, E and F). The survival of CT2A tumor–bearing LGMN-mKD mice was significantly extended when compared with that of control mice (Figure 2I). The extended survival and reduced macrophage infiltration observed in CT2A-bearing LGMN-mKD mice were negated by the treatment with Colivelin (Figure 2, I–K). However, Colivelin treatment alone did not affect the survival of CT2A tumor–bearing mice (Figure 2I), as well as GBM cell proliferation and apoptosis in vitro (Supplemental Figure 3, G–J) and in vivo (Supplemental Figure 3, K and L). Together, these findings reveal that TAM LGMN promotes macrophage infiltration and GBM progression by activating the GSK3β/STAT3 axis.

### TAM-derived LGMN regulates GBM cell proliferation and apoptosis.

**Figure 3:**
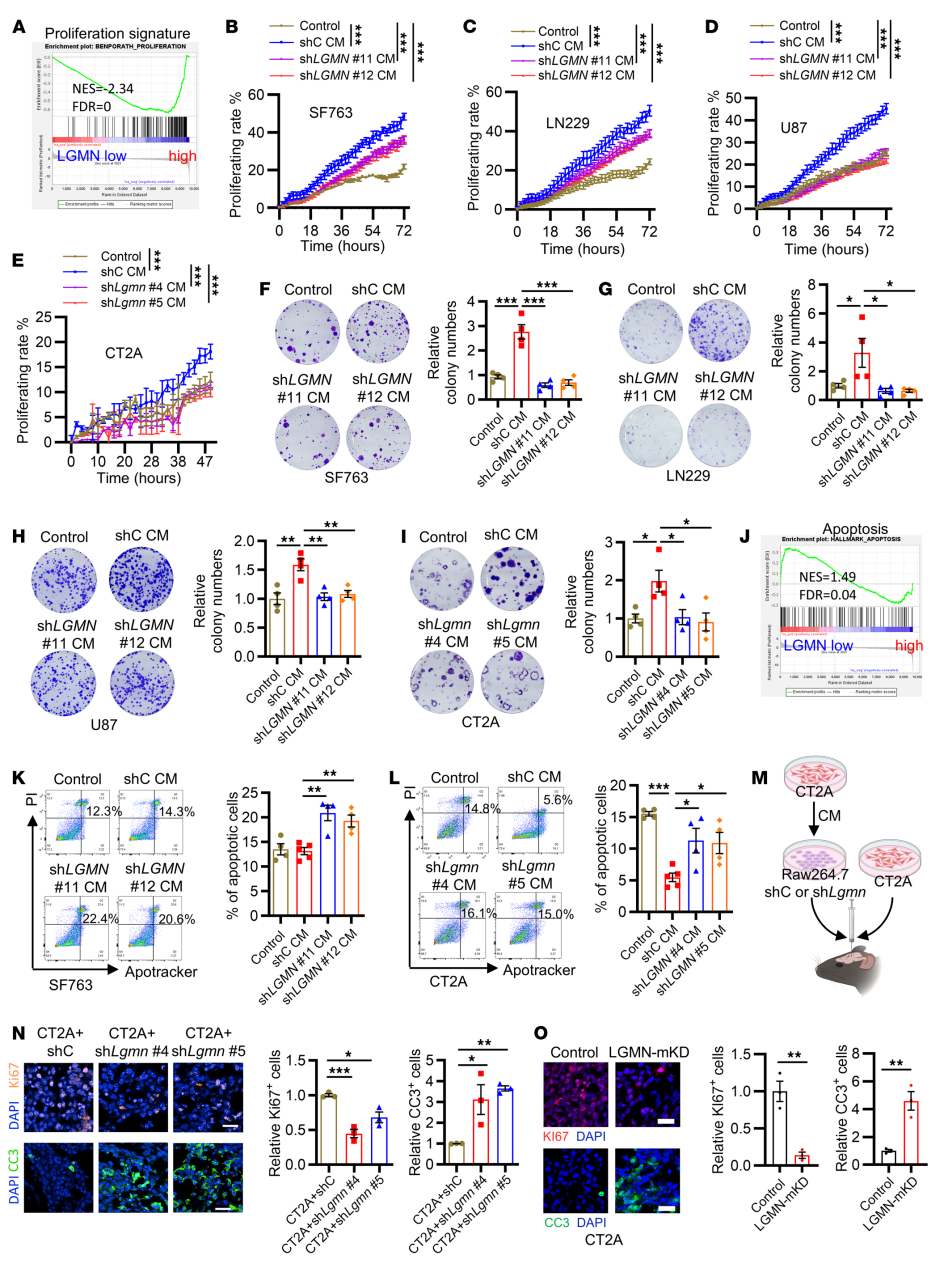
Macrophage-derived LGMN regulates GBM cell proliferation and apoptosis. (**A**) GSEA shows enrichment of proliferation signature in tumors from the *LGMN*-low compared with the *LGMN*-high macrophage group. Normalized enrichment score (NES) and FDR *q* values are shown. (**B**–**D**) Proliferation curves of SF763 (**B**), LN229 (**C**), and U87 (**D**) cells treated with CM from THP1 macrophages expressing shRNA control (shC) and *LGMN* shRNAs (sh*LGMN*). GBM cell proliferation was recorded and analyzed using the Incucyte imaging system for 72 hours. *n* = 6 independent samples. (**E**) Proliferation curves of CT2A cells treated with CM from Raw264.7 macrophages expressing shC and sh*Lgmn*. CT2A GBM cell proliferation was recorded and analyzed using the Incucyte imaging system for 48 hours. *n* = 6 independent samples. (**F**–**I**) Colony formation assay and quantifications showing proliferation of SF763 (**F**), LN229 (**G**), U87 (**H**), and CT2A (**I**) cells treated with CM from THP1 or Raw264.7 macrophages expressing shC and sh*LGMN*. *n* = 4 independent samples. (**J**) GSEA shows enrichment of apoptosis signature in the *LGMN*-low compared with the *LGMN*-high macrophage group. NES and FDR *q* values are shown. (**K** and **L**) Representative images and quantification of Apotracker and propidium iodide (PI) staining showing apoptosis of SF763 (**K**) and CT2A (**L**) cells treated with CM from THP1 or Raw264.7 macrophages expressing shC and sh*LGMN*. *n* = 4 independent samples. (**M**) Diagram showing procedures of coinjection of CT2A cells and CT2A CM–educated Raw264.7 macrophages harboring shC or sh*Lgmn* into brains of C57BL/6 mice. (**N**) Representative images and quantification of IF for relative expression of Ki67 and cleaved caspase-3 (CC3) in size-matched tumors from C57BL/6 mice implanted with CT2A cells and CT2A CM–polarized Raw264.7 macrophages expressing shC or sh*Lgmn*. Scale bars: 25 μm. *n* = 3 independent samples. (**O**) Representative images and quantification of IF for relative expression of Ki67 and CC3 in size-matched tumors from control and LGMN–macrophage-specific knockdown (LGMN-mKD) mice implanted with CT2A cells. Scale bars: 25 μm. *n* = 3 independent samples. Two-way ANOVA test (**B**–**E**); 1-way ANOVA test (**F**–**I**, **K**, **L**, **N**, and **O**). *P < 0.05, **P < 0.01, ***P < 0.001.

To elucidate the role of TAM-derived LGMN in GBM cells, we compared the scRNA-Seq profiles (Gene Expression Omnibus [GEO] GSE182109) of GBM cells from patients who harbored tumors with TAMs expressing high and low *LGMN*. GSEA results demonstrated that cancer cell proliferation signatures were the top hits downregulated in tumors with low *LGMN* expression in TAMs (Figure 3A and Supplemental Figure 4, A and B), suggesting a connection between TAM-derived LGMN and cancer cell proliferation in GBM. To assess the impact of LGMN on GBM cell proliferation, SF763, LN229, U87, and CT2A cells were exposed to LGMN recombinant protein at distinct concentrations. The Incucyte proliferation assays showed that LGMN recombinant protein promoted GBM cell proliferation in a dose-dependent manner (Supplemental Figure 4, C–F). To further determine whether macrophage-derived LGMN could affect GBM cell proliferation, GBM cells (e.g., SF763, LN229, U87, and CT2A cells) were treated with the CM collected from THP1 and Raw264.7 macrophages with or without *LGMN* knockdown. Incucyte proliferation assays demonstrated that THP1 and Raw264.7 CM promoted GBM cell proliferation, and this promotion was abolished by shRNA-mediated depletion of LGMN (Figure 3, B–E) or pharmacologic inhibition of LGMN with C11 and RR-11a (Supplemental Figure 4, G–J) in macrophages. Additionally, the colony formation assay results demonstrated that the CM from *LGMN*-depleted macrophages (including THP1 and Raw264.7) decreased the number of colonies formed by SF763, LN229, U87, and CT2A cells (Figure 3, F–I).

In contrast to proliferation, apoptosis signature was significantly enriched in cancer cells from tumors harboring low *LGMN* expression in TAMs (Figure 3J), indicating that LGMN might support GBM cell survival. Indeed, flow cytometry assays showed that LGMN recombinant protein inhibited the apoptosis of SF763, LN229, U87, and CT2A cells in a dose-dependent manner (Supplemental Figure 4K). Moreover, compared with the CM from shRNA control macrophages (including THP1 and Raw264.7 macrophages), the CM from *LGMN*-depleted macrophages promoted apoptosis of SF763 and CT2A cells (Figure 3, K and L). To validate the effect of macrophage-derived LGMN on GBM cell biology in vivo, we co-implanted CT2A cells and TAMs (Raw264.7 macrophages pretreated with GBM cell CM) into the brains of C57BL/6 mice (Figure 3M). The results showed that co-implantation of CT2A and polarized macrophages harboring LGMN shRNA significantly decreased proliferation and enhanced apoptosis compared with polarized macrophages harboring shRNA control in GBM tumors (Figure 3N). Similarly, the reduced proliferation and enhanced apoptosis were observed in tumors from LGMN-mKD mice compared with control mice (Figure 3O). Together, these findings suggest that macrophage-derived LGMN could directly regulate GBM cell proliferation and apoptosis in vitro and in vivo.

### LGMN regulates GBM cell proliferation and apoptosis through integrin α_v_.

**Figure 4:**
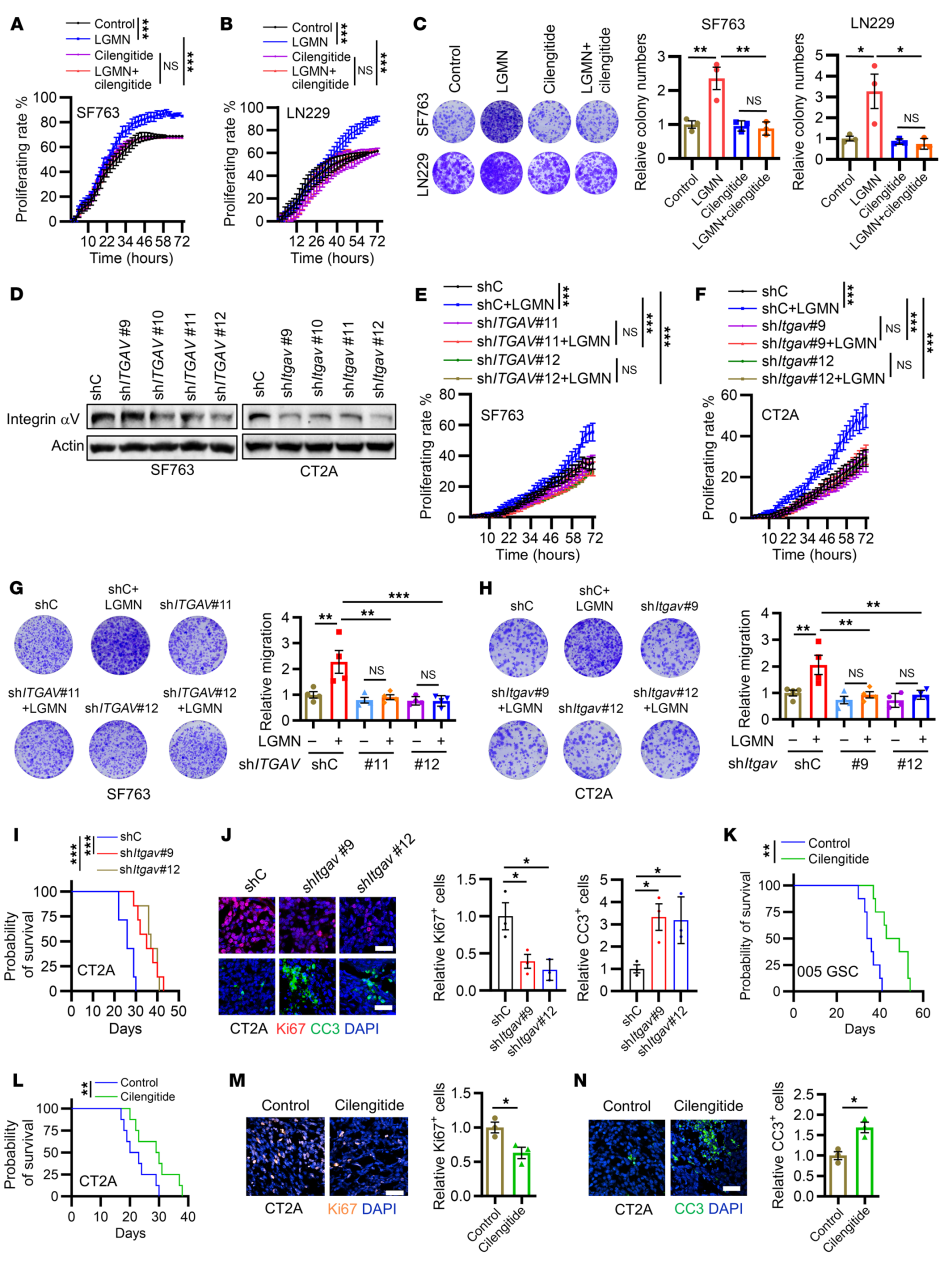
Integrin α_v_ is essential for LGMN-induced GBM cell proliferation. (**A** and **B**) Incucyte proliferation curves of SF763 (**A**) and LN229 (**B**) cells incubated with LGMN recombinant protein (100 ng/mL) in the presence or absence of integrin α_v_ inhibitor cilengitide (6.5 μg/mL for SF763 and 10 μg/mL for LN229). (**C**) Colony formation assay shows proliferation of SF763 and LN229 cells incubated with LGMN protein in the presence or absence of cilengitide. (**D**) Immunoblots for integrin α_v_ in lysates of SF763 and CT2A cells expressing shRNA control (shC) and *ITGAV* shRNA (sh*ITGAV*). (**E** and **F**) Incucyte proliferation curves of shC and sh*ITGAV*-transfected SF763 (**E**) and CT2A (**F**) cells treated with or without LGMN protein. (**G** and **H**) Colony formation assay shows proliferation of shC and sh*ITGAV*-transfected SF763 (**G**) and CT2A (**H**) cells incubated with or without LGMN protein. (**I**) Survival curves of C57BL/6 mice implanted with 2 × 10^4^ shC and sh*Itgav* CT2A cells. *n* = 7 mice per group. (**J**) Representative images and quantification of IF for relative expression of Ki67 and CC3 in size-matched shC and sh*Itgav* CT2A tumors. Scale bars: 25 μm. (**K** and **L**) Survival curves of C57BL/6 mice implanted with 2 × 10^5^ 005 GSC (**K**) or 2 × 10^4^ CT2A cells (**L**) and treated with cilengitide (30 mg/kg, i.p., daily). *n* = 8 mice per group. (**M** and **N**) Representative images and quantification of IF for relative expression of Ki67 (**M**) and CC3 (**N**) in size-matched CT2A tumors from C57BL/6 mice treated with cilengitide. Scale bars: 25 μm. *n* = 3 (**C**, **G**, **H**, and **J**) or 6 (**A**, **B**, **E**, and **F**) independent samples. Two-way ANOVA test (**A**, **B**, **E**, and **F**); 1-way ANOVA test (**C**, **G**, **H**, **J**, and **M**); log-rank test (**I**, **K**, and **L**); Student’s *t* test (**M** and **N**). *P < 0.05, **P < 0.01, ***P < 0.001.

Given the potential interaction between LGMN and integrin α_v_ in vascular smooth muscle cells (56), we hypothesized that integrin α_v_ may be required for eliciting the regulatory function of LGMN in GBM cells. Bioinformatics analyses demonstrated that in addition to TAMs, cancer cells (CD45^–^ cells) highly expressed integrin α_v_, which positively correlated with LGMN expression in patients from the Chinese Glioma Genome Atlas (CGGA) GBM database (Supplemental Figure 5, A and B). To explore the potential role of integrin α_v_ in mediating LGMN-driven proliferation of GBM cells, we first optimized the concentration of the integrin α_v_ inhibitor cilengitide by plotting the dose-response curves in SF763, LN229, U87, and CT2A cells. Since each GBM cell has a different response to cilengitide, we aimed to identify the concentrations that would minimally affect the growth of each GBM cell line, setting a threshold response of no more than 20% (Supplemental Figure 5, C–F). The Incucyte live imaging system was used to track GBM cell proliferative activity upon the treatment with cilengitide with the optimized concentrations. The results showed that cilengitide treatment negated LGMN-induced proliferation upregulation in SF763, LN229, U87, and CT2A cells (Figure 4, A and B, and Supplemental Figure 5, G and H). Similarly, such an effect was apparent in colony formation assays (Figure 4C and Supplemental Figure 5I). Next, we used a shRNA-mediated knockdown system to deplete integrin α_v_ (encoded by *ITGAV*) in SF763 and CT2A cells (Figure 4D) and found that LGMN-induced upregulation of GBM cell proliferation (Figure 4, E and F) and colony formation (Figure 4, G and H) were abolished by integrin α_v_ depletion. Finally, we found that LGMN-induced survival support for GBM cells was rescued by pharmacologic (Supplemental Figure 5J) and genetic (Supplemental Figure 5K) inhibition of integrin α_v_. To confirm these findings in vivo, we implanted control and integrin α_v_–depleted CT2A cells into mouse brains and found that integrin α_v_ depletion extended survival (Figure 4I), decreased proliferation, and increased apoptosis in tumors (Figure 4J). Additionally, we treated CT2A and 005 GSC tumor–bearing mice with cilengitide and found that such treatment significantly prolonged the survival of tumor-bearing mice (Figure 4, K and L). IF staining showed that cilengitide treatment led to lower proliferation and higher apoptosis in CT2A tumors (Figure 4, M and N). Together, these findings highlight that LGMN regulates GBM cell biology via integrin α_v_ signaling.

### LGMN regulates GBM cell biology by activating AKT and p65 pathways.

**Figure 5:**
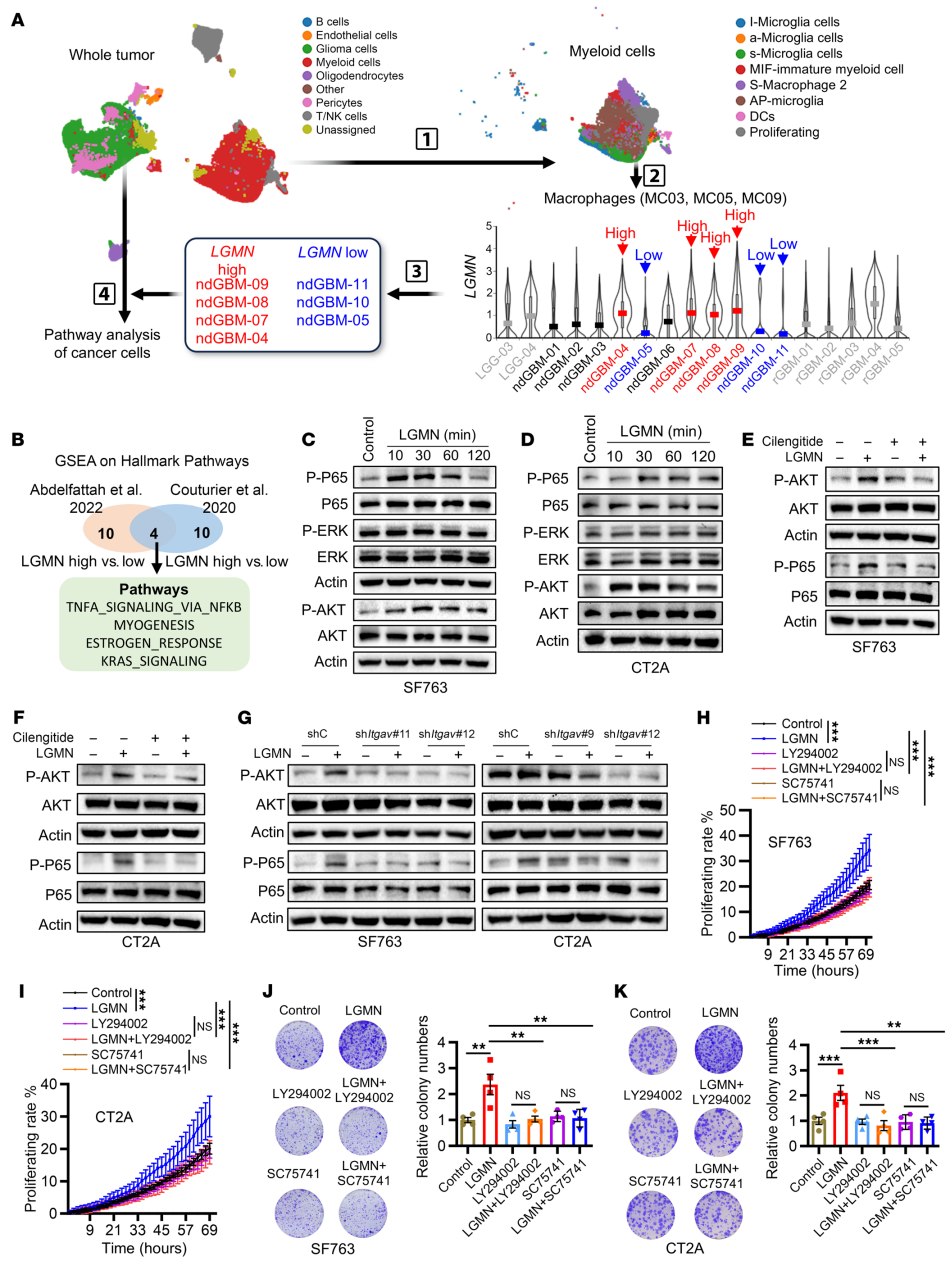
LGMN promotes GBM cell proliferation by activating AKT and p65 pathways. (**A**) Workflow for identifying pathways in cancer cells that are regulated by macrophage-derived LGMN. scRNA-Seq profiles of myeloid cells were subclustered from all cell populations and then macrophage subclusters were annotated. Based on *LGMN* expression in macrophages, newly diagnosed GBM patients were further classified as *LGMN*-high and *LGMN*-low groups. GSEA was performed to compare scRNA-Seq profiles of cancer cells extracted from *LGMN*-high and -low groups. (**B**) Identification of 4 hallmark pathways (as indicated) in cancer cells from distinct scRNA-Seq datasets with the same strategy (macrophage *LGMN* high vs. *LGMN* low). (**C** and **D**) Immunoblots for p-p65, p65, p-ERK, ERK, p-AKT, and AKT in cell lysates of SF763 (**C**) and CT2A (**D**) cells treated with LGMN recombinant protein (100 ng/mL) for indicated times. (**E** and **F**) Immunoblots for p-p65, p65, p-AKT, and AKT in cell lysates of SF763 (**E**) and CT2A (**F**) cells treated with LGMN protein in the presence or absence of integrin α_v_ inhibitor cilengitide (25 μg/mL). (**G**) Immunoblots for p-p65, p65, p-AKT, and AKT in cell lysates of SF763 and CT2A cells expressing shRNA control (shC) and *ITGAV* shRNA (sh*ITGAV*) and treated with or without LGMN protein for 30 minutes. (**H** and **I**) Proliferation curves of SF763 (**H**) and CT2A (**I**) cells incubated with LGMN protein in the presence or absence of AKT inhibitor LY294002 (2.5 μmol/L for SF763 and 0.8 μmol/L for CT2A) or p65 inhibitor SC75741. *n* = 6 independent samples. (**J** and **K**) Colony formation assay shows proliferation of SF763 (**J**) and CT2A (**K**) cells incubated with LGMN protein in the presence or absence of LY294002 or SC75741. *n* = 4 independent samples. Two-way ANOVA test (**H** and **I**); 1-way ANOVA test (**J** and **K**). **P < 0.01, ***P < 0.001.

To reveal the potential downstream of the LGMN/integrin α_v_ axis in GBM cells, we comprehensively analyzed scRNA-Seq data (GSE182109) from newly diagnosed GBM patient tumors. The myeloid cell populations were subclustered into different subpopulations, including microglia (MC01, MC02, and MC06), macrophages (MC03, MC05, and MC09), dendritic cells (MC08), and MIF-immature myeloid cells (MC04), using a previously reported annotation method (29). According to *LGMN* expression in macrophage subclusters (MC03, MC05, and MC09), newly diagnosed GBM patients were further subclassified into *LGMN*-high and -low groups. GSEA was performed on scRNA-Seq profiles of GBM cells from these 2 groups to identify the key signaling pathways in GBM cells that are potentially regulated by macrophage-derived LGMN (Figure 5A). Additionally, we used the same approach to analyze another scRNA-Seq dataset (EGAS00001004422) from newly diagnosed IDH-WT GBM patient tumors. As a result, we identified 4 overlapping pathways (TNF-α signaling via NF-κB, myogenesis, estrogen response, and KRAS signaling) that were highly enriched in the *LGMN*-high group (Figure 5B). Western blotting results demonstrated that LGMN did not affect the expression of ERα, ERβ, and PAX3, a key regulator of myogenesis (57–59), in SF763 cells (Supplemental Figure 6A). These results led us to focus on investigating whether LGMN could regulate KRAS signaling and the NF-κB pathway in GBM cells. There are 2 major downstream pathways of KRAS signaling: the MAPK and PI3K pathways (60, 61), which are characterized by the phosphorylation of ERK and AKT, respectively (Supplemental Figure 6B). Western blotting validations demonstrated that LGMN recombinant protein upregulated the phosphorylation of NF-κB p65 (p-p65) and AKT (p-AKT), but not ERK (p-ERK), in SF763, CT2A, LN229, and U87 cells (Figure 5, C and D, and Supplemental Figure 6, C and D). To confirm this observation in vivo, we used flow cytometry to analyze p-AKT and p-p65 in CD45^–^CD11b^–^ GBM cells isolated from CT2A tumors (Supplemental Figure 6E). The results showed that p-AKT and p-p65 in CD45^–^CD11b^–^ GBM cells were reduced by LGMN depletion in macrophages (Supplemental Figure 6, F and G), supporting that macrophage-derived LGMN activates AKT and p65 in GBM cells.

Given that integrin α_v_ is the receptor of LGMN on GBM cells, we hypothesized that p65 and AKT are downstream of integrin α_v_ and are required for the effects of LGMN on GBM cell biology. Western blotting validations demonstrated that LGMN-induced upregulation of p-p65 and p-AKT in SF763, CT2A, LN229, and U87 cells was abolished by treatment with the integrin α_v_ inhibitor cilengitide (Figure 5, E and F, and Supplemental Figure 6, H and I). Similarly, shRNA-mediated depletion of integrin α_v_ blocked the promotion of p-p65 and p-AKT by LGMN in GBM cells (Figure 5G). Consistent with our in vitro observation, mice bearing integrin α_v_–depleted tumors exhibited lower p-p65 and p-AKT in CD45^–^CD11b^–^ cancer cells (Supplemental Figure 6, J and K). To reveal the relationship between p65 and AKT, GBM cells were treated with LGMN in the presence or absence of the NF-κB p65 pathway inhibitor SC75741 or the PI3K/AKT pathway inhibitor LY294002. Consistent with previous studies showing that AKT regulates the activity of p65 (62–64), we found that inhibition of AKT using LY294002 abolished LGMN-driven p-p65 in GBM cells (Supplemental Figure 6, L–O). However, LGMN-induced upregulation of p-AKT was partially rescued or not affected by treatment with SC75741 in SF763, U87, LN229, and CT2A cells (Supplemental Figure 6, P–S). To investigate whether LGMN-induced GBM cell proliferation is regulated by the AKT/p65 pathway, we first optimized the concentration of the AKT inhibitor LY294002 and the p65 inhibitor SC75741 in SF763, LN229, U87, and CT2A cells (Supplemental Figure 7, A–H). Using an optimized concentration of each compound that does not directly affect GBM cell growth (Supplemental Figure 7, A–H), we performed proliferation assays using Incucyte live imaging and colony formation. The results showed that inhibition of the NF-κB p65 pathway or the PI3K/AKT pathway abolished the pro-proliferating effect of LGMN on SF763, LN229, U87, and CT2A cells (Figure 5, H–K, and Supplemental Figure 7, I–L). Flow cytometry analyses demonstrated that inhibition of the NF-κB p65 pathway or the PI3K/AKT pathway negated the pro-survival (decreased apoptosis) effect of LGMN on SF763, LN229, U87, and CT2A cells (Supplemental Figure 8, A–D). Together, these findings highlight a critical role of the integrin α_v_/AKT/p65 signaling pathway in mediating LGMN-induced GBM cell proliferation and survival.

### Inhibition of GSK3β, STAT3, and integrin α_v_ synergizes with anti–PD-1 therapy.

**Figure 6:**
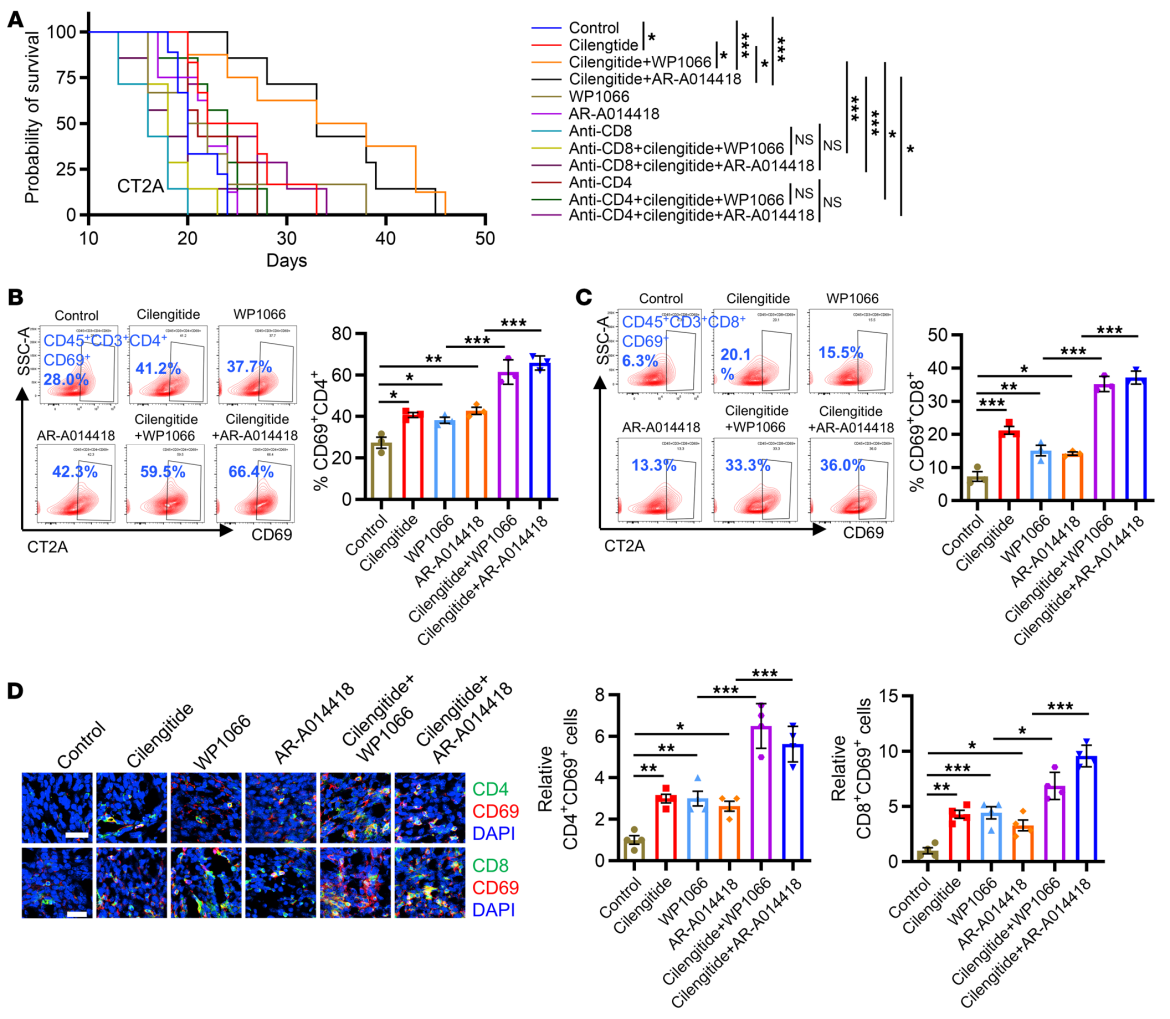
Combined inhibition of integrin α_v_ and GSK3β or STAT3 activates antitumor immunity. (**A**) Survival curves of C57BL/6 mice implanted with CT2A cells. Mice were treated with integrin α_v_ inhibitor cilengitide (30 mg/kg, i.p., daily), STAT3 inhibitor WP1066 (30 mg/kg, i.p., daily), and GSK3β inhibitor AR-A014418 (30 mg/kg, i.p., daily). For T cell depletion, anti-CD8 (Bio X Cell, BE0061, clone 2.43) or anti-CD4 (Bio X Cell, BE003-1, clone GK1.5) antibodies were injected intraperitoneally (300 mg per mouse) starting on day 2 after tumor injection for 3 consecutive days and every 5 days thereafter. *n* = 6–8 mice per group. (**B** and **C**) Representative images and quantification of flow cytometry for percentage of CD45^+^CD3^+^CD4^+^CD69^+^ cells (**B**) and CD45^+^CD3^+^CD8^+^CD69^+^ cells (**C**) in tumor tissues from size-matched CT2A tumor–bearing C57BL/6 mice. *n* = 3 independent samples. (**D**) Representative images and quantification of IF analysis for CD4^+^CD69^+^ and CD8^+^CD69^+^ cells in size-matched CT2A tumors from C57BL/6 mice. *n* = 4 independent samples. Scale bars: 25 μm. Log-rank test (**A**); 1-way ANOVA test (**B**–**D**). *P < 0.05, **P < 0.01, ***P < 0.001.

Given the dual mechanism of LGMN for regulating macrophage infiltration and GBM cell proliferation through activating GSK3β/STAT3 and integrin α_v_/AKT/p65 signaling, respectively, we explored the impact of cotargeting macrophages (using the GSK3β inhibitor AR-A014418 or the STAT3 inhibitor WP1066) and GBM cells (using the integrin α_v_ inhibitor cilengitide) in mouse models. In GSC272 (a patient-derived xenograft) GBM tumors implanted in immunocompromised nude mice, we found that cilengitide alone prolonged the survival of tumor-bearing mice; however, the combination of cilengitide with WP1066 or AR-A014418 did not further offer survival benefit (Supplemental Figure 9A). In CT2A tumors implanted in immunocompetent C57BL/6 mice, cilengitide treatment extended the survival, and this effect was further amplified when it was combined with WP1066 or AR-A014418 (Figure 6A). These in vivo findings suggest that the immune system is required for the antitumor effect of cotargeting GBM cells and macrophages. Indeed, depletion of CD8^+^ or CD4^+^ T cells abolished the survival extension of CT2A tumor–bearing mice induced by the treatment with cilengitide in combination with WP1066 or AR-A014418 (Figure 6A). Flow cytometry analysis of splenic T cells from CT2A tumor–bearing mice demonstrated that treatment with cilengitide, AR-A014418, or WP1066 increased the populations of CD3^+^ (CD45^+^CD3^+^) and CD8^+^ (CD45^+^CD3^+^CD8^+^CD4^–^) T cells, but not CD4^+^ (CD45^+^CD3^+^CD4^+^CD8^–^) T cells (Supplemental Figure 8, B–D). Combination of cilengitide with AR-A014418 or WP1066 further increased CD3^+^ and CD8^+^ T cells, but not CD4^+^ T cells (Supplemental Figure 9, B–D). Furthermore, cilengitide, WP1066, or AR-A014418 treatment increased activated CD4^+^ (CD45^+^CD3^+^CD8^–^CD4^+^CD69^+^) and CD8^+^ T cells (CD45^+^CD3^+^CD8^+^CD4^–^CD69^+^), and these effects were improved when CT2A tumor–bearing mice received the treatment with cilengitide combined with WP1066 or AR-A014418 (Supplemental Figure 9, E and F). Similarly, tumor-infiltrating CD3^+^, CD8^+^, activated CD4^+^, and activated CD8^+^ T cells, but not CD4^+^ T cells, were upregulated by treatment with cilengitide, AR-A014418, or WP1066 (Figure 6, B and C, and Supplemental Figure 10, A–D). The enhancement was amplified further when cilengitide was combined with AR-A014418 or WP1066 (Figure 6, B and C, and Supplemental Figure 10, A–D). In addition, the enhanced frequency of activated CD4^+^ and CD8^+^ T cells was confirmed by IF staining in CT2A tumors (Figure 6D).

**Figure 7:**
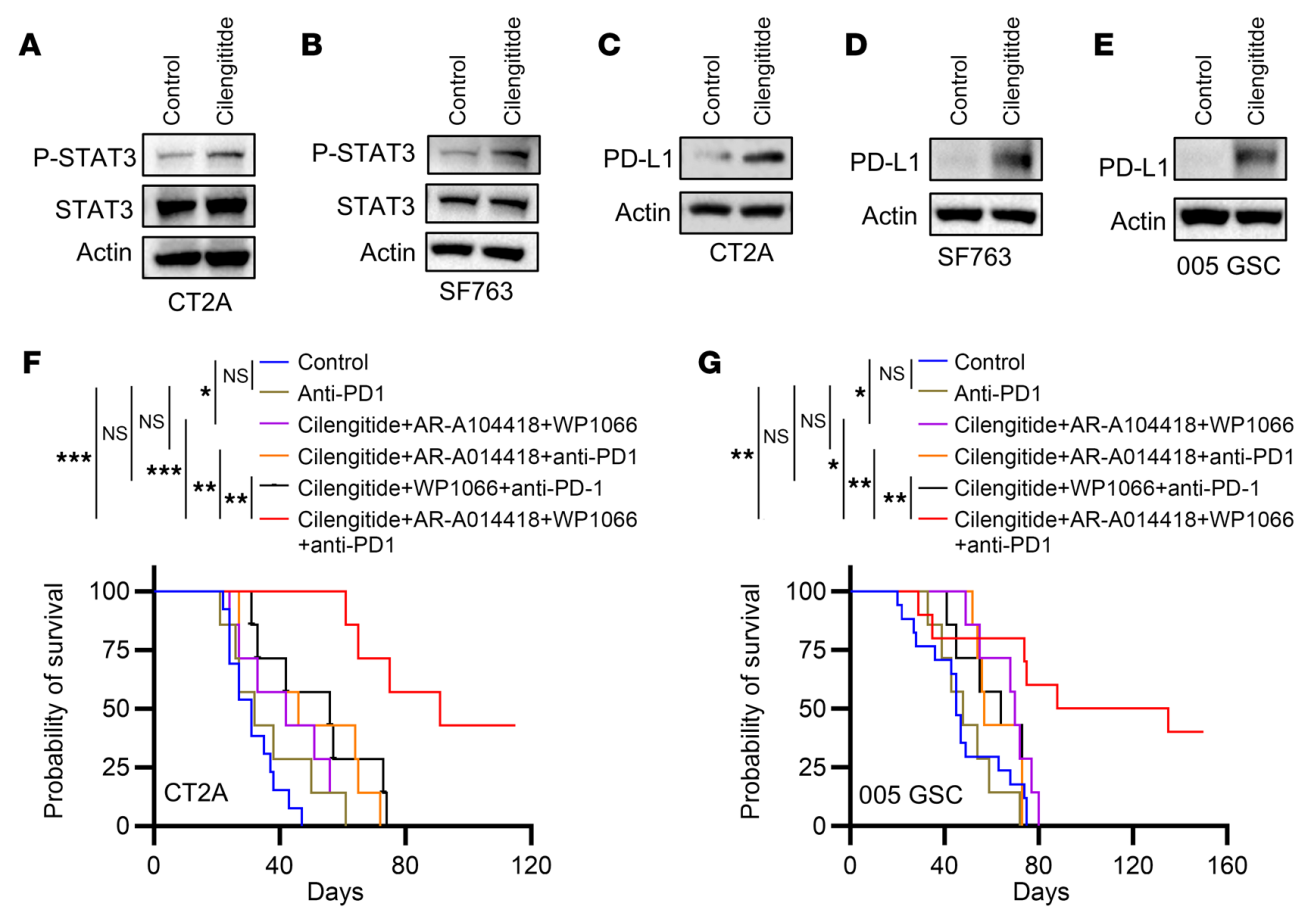
Inhibition of integrin α_v_, GSK3β, and STAT3 synergizes with anti–PD-1 therapy. (**A** and **B**) Immunoblots for p-STAT3 and STAT3 in cell lysates of CT2A (**A**) and SF763 (**B**) cells treated with or without cilengitide (25 μg/mL) for 1 hour. (**C**–**E**) Immunoblots for PD-L1 in cell lysates of CT2A (**C**), SF763 (**D**), and 005 GSC (**E**) cells treated with or without cilengitide (25 μg/mL) for 24 hours. (**F** and **G**) Survival curves of C57BL/6 mice implanted with CT2A cells (**F**) and 005 GSC cells (**G**). Mice were treated with cilengitide (30 mg/kg, i.p., daily), WP1066 (30 mg/kg, i.p., daily), and AR-A014418 (30 mg/kg, i.p., daily) starting on day 7 and then anti–PD-1 (Bio X Cell, BE0146, clone RMP1-14; 10 mg/kg, i.p.) on days 11, 14, and 17. *n* = 6–14 mice per group. Log-rank test (**F** and **G**). *P < 0.05, **P < 0.01, ***P < 0.001.

Given the relationship between cilengitide treatment and STAT3 activation observed in melanoma cells (65), we performed Western blotting experiments showing that cilengitide treatment upregulated p-STAT3 in GBM cells (Figure 7, A and B), suggesting that dual targeting of integrin α_v_ and STAT3 is required for blocking LGMN-induced GBM biology. Consistent with previous studies showing that STAT3 could induce PD-L1 expression in various cancer cells (66–68), we found that cilengitide treatment enhanced the expression of PD-L1 in GBM cells (Figure 7, C–E). These findings prompted us to investigate the antitumor effect of cotargeting GBM cells (using the integrin α_v_ inhibitor cilengitide combined with the STAT3 inhibitor WP1066) and macrophages (using the GSK3β inhibitor AR-A014418 or the STAT3 inhibitor WP1066) combined with anti–PD-1 therapy in GBM-bearing mice. Our results demonstrated that the combination therapy (cilengitide combined with WP1066 or AR-A014418) did not affect the antitumor efficiency of anti–PD-1 therapy in CT2A and 005 GSC GBM mouse models (Figure 7, F and G). However, the triple therapy with cilengitide, WP1066, and AR-A014418 synergized with anti–PD-1 therapy to generate a complete tumor regression in 42%–50% of CT2A and 005 GSC tumor–bearing mice (Figure 7, F and G). Together, these findings suggest that targeting of LGMN-mediated macrophage-GBM interactions combined with anti–PD-1 therapy is a promising therapeutic strategy for GBM.

## Discussion

In this study, we explored the mechanisms of the pro-tumor effect of LGMN, a key protease that is highly expressed by TAMs, the most abundant cells in GBM tumor mass, accounting for up to 50% of its total cells (14, 15, 17, 69). By integrating scRNA-Seq analysis and functional studies, we demonstrated that LGMN promotes GBM progression via a mechanism of dual regulation of macrophages and GBM cells. Specifically, LGMN intrinsically promotes macrophage infiltration by activating the GSK3β/STAT3 axis, whereas TAM-derived LGMN regulates GBM cell proliferation and apoptosis through the integrin α_v_/AKT/p65 axis. Simultaneous targeting of LGMN-triggered downstream signaling pathways exhibited a gained benefit and synergized with anti–PD-1 therapy in GBM mouse models. Together, our work reveals the role and underlying mechanism of LGMN-mediated tumor-macrophage interaction and supports the effort to develop therapeutic strategy by dually targeting tumor-macrophage symbiosis and immune checkpoints in GBM.

LGMN plays various roles in mammalian physiology and immunology (40, 70–73). Recent evidence demonstrates that LGMN is highly expressed in macrophages (37, 74–77) and can be upregulated under pathological conditions (37, 41, 74, 78, 79), such as myocardial infarction surgery and high-fat diet–induced obesity (74). The increased macrophage LGMN contributes to disease progression and/or tissue repair through a context-dependent mechanism. For example, pulmonary macrophage–derived LGMN promotes hypertension by activating MMP-2/TGF-β1 signaling in pulmonary artery smooth muscle cells (78). Adipose tissue macrophage–derived LGMN upregulates inflammatory responses and exacerbates obesity development by attenuating PKA activation in adipocytes (79). LGMN derived from cardiac resident macrophages improves cardiac repair by clearing apoptotic cardiomyocytes (74). Our previous (37) and current studies highlight the role of TAM-derived LGMN in GBM progression and immunosuppression, suggesting that LGMN is a promising target for GBM immunotherapy. Our preclinical studies have shown that LGMN inhibition in combination with anti–PD-1 therapy can inhibit tumor progression, but not cure any tumor-bearing mice (37), suggesting that further efforts are needed to reveal the molecular basis underlying this therapy resistance and develop effective LGMN-targeted/related therapies.

LGMN has been well recognized for its role in promoting tumor progression through distinct mechanisms in various types of cancers (39–41, 80). In GBM, LGMN can promote tumor progression by downregulating the p53 protein (81). In addition, LGMN can cleave DEAD-box helicase 3 X-linked (DDX3X), facilitating adaptation of GBM cells to hypoxia and nutrient-deprived TME by inducing alternative RNA splicing events (41). Given that the AKT pathway can regulate p53 protein stability and DDX3X phosphorylation (82–85), it is plausible that AKT is involved in LGMN-induced GBM cell proliferation. This hypothesis is supported by our results in the current study showing that LGMN can activate the AKT/p65 pathway to promote GBM cell proliferation and survival, and by previous studies in epithelial ovarian carcinoma, gastric carcinoma, and breast cancer showing that LGMN stimulates tumor growth and progression via activating AKT pathways (86–88). Further studies are required to determine whether p53 and DDX3X signals are involved in LGMN/AKT/p65 axis–directed GBM cell biology.

In exploring the connection between LGMN and the AKT/p65 signaling axis, we observed that integrin α_v_ is the receptor of LGMN on GBM cells that mediate LGMN’s function via activation of a downstream pro-tumor signaling axis, consistent with previous work (56). However, further studies are needed to investigate whether other LGMN receptors, such as TLRs and integrin α_5_β_1_ (86, 89), exist in the GBM system, and if so, how they mediate this context-dependent TAM-tumor symbiosis. Although our preclinical findings from GBM mouse models support an effort to develop integrin α_v_–targeted therapy, it is well accepted that targeted therapy against specific signaling pathways in GBM cells has not been successful in clinical trials owing to GBM cell heterogeneity and the compensatory change of pro-tumor signals upon treatments (13, 14, 17, 33, 90). This hypothesis is supported by the results from a phase III clinical trial (ClinicalTrials.gov NCT00689221) showing that cilengitide treatment did not achieve the desired antitumor efficacy in newly diagnosed GBM patients when combined with radiotherapy (91). In this study, we observed that cilengitide treatment in GBM induces activation of STAT3, which plays an important role in promoting GBM progression, GSC stemness, and immunosuppression by regulating PD-L1 expression (19). In the current study, we offer an alternative strategy that may improve the effectiveness of cilengitide for GBM, given that our preclinical trials demonstrated that dual targeting of integrin α_v_ (using cilengitide) and STAT3 (using WP1066) generates a potent antitumor effect in GBM mouse models. A previous study has shown that WP1066 enhances the effectiveness of whole-brain radiotherapy in an immune-competent GBM mouse model (92). The synergistic effect of WP1066 and radiotherapy is likely due to the induction of interactions between dendritic cells and T cells in the GBM TME (92). Since treatment with cilengitide or AR-A014418 increases T cell infiltration and activation in GBM, these two inhibitors may further enhance the synergistic effect of WP1066 and radiotherapy for GBM patients.

In addition to cancer cell biology, LGMN may regulate the TME (40). Together with our recent findings (37, 77), the current study uncovers that LGMN could sustain an immunosuppressive TME by upregulating TAM infiltration and immunosuppressive polarization in GBM by activating the GSK3β/STAT3 axis. This mechanism is consistent with a previous study showing that LGMN increases endothelial barrier permeability via STAT3 signaling (93). Despite the importance of the GSK3β/STAT3 axis in this process, we observed that inhibition of GSK3β or STAT3 signaling does not generate valuable survival benefits in GBM tumor–bearing mice. Given the contribution of tumor-macrophage symbiosis in promoting GBM progression (14, 15, 17, 20), we further developed combination therapy simultaneously targeting LGMN-induced effects on GBM cells (integrin α_v_ and STAT3) and macrophages (GSK3β or STAT3) and observed a potent antitumor activity in immunocompetent GBM mouse models. GBM is a typical “immune-cold” tumor with a scarcity of T cells and high infiltration of TAMs (14, 15, 94). We demonstrated that inhibition of tumor-macrophage symbiosis via blockade of integrin α_v_ combined or not combined with GSK3β or STAT3 inhibition promotes the infiltration and activation of T cells, especially CD8^+^ T cells, in the GBM TME. In addition to functioning as an LGMN receptor on GBM cells, integrin α_v_ has been shown to be an osteopontin receptor on macrophages and a TFPI2 receptor on microglia to mediate their polarization toward an immunosuppressive phenotype (24, 25); cilengitide may also inhibit macrophage and microglia immunosuppressive polarization in the GBM TME, thus further activating CD8^+^ T cell–mediated antitumor immunity and enhancing antitumor efficiency of immunotherapies. Indeed, the study results presented here demonstrated that simultaneous inhibition of integrin α_v_, STAT3, and GSK3β using cilengitide, WP1066, and AR-A014418, respectively, synergizes with anti–PD-1 therapy and offers a complete tumor regression in about 50% of GBM tumor–bearing mice. Together, our findings highlight that targeting of LGMN-directed tumor-macrophage symbiosis coupled with anti–PD-1 therapy is a promising combination strategy.

## Methods

### Sex as a biological variable.

Our study examined 6-week-old female athymic mice (J:NU) and C57BL/6 mice, which were purchased from The Jackson Laboratory and housed under aseptic conditions. The animals are well established and were used to develop orthotopic GBM models as described in our published studies (22, 22–24, 37, 77). There are no reported sex differences among GBM patients with LGMN-high and LGMN-low TAMs. Sex was not considered as a biological variable in this study.

### Cell culture.

THP1, U937, and Raw264.7 cells were cultured in RPMI 1640 medium (RPMI) containing 1:100 antibiotic-antimycotic (Gibco, 15140-122) and 10% fetal bovine serum (FBS; Thermo Fisher Scientific, 16140071). THP1 and U937 cells were differentiated into macrophages by administration of 200 ng/mL phorbol 12-myristate 13-acetate (Sigma-Aldrich) for 24 hours. SF763, LN229, U87, 293T, and CT2A cells were cultured in Dulbecco’s modified Eagle medium (DMEM; Gibco, 11995-065) with 1:100 antibiotic-antimycotic and 10% FBS. 005 GSC and GSC272 tumors were provided by Samuel D. Rabkin (Massachusetts General Hospital, Boston, Massachusetts, USA) and Frederick F. Lang (MD Anderson Cancer Center, Houston, Texas, USA), respectively, and cultured in neural stem cell (NSC) proliferation medium (Millipore, SCM005) containing 20 ng/mL bFGF (PeproTech, 100-18B) and EGF (PeproTech, AF-100-15). Other cells were purchased from the American Type Culture Collection. All cells were validated as mycoplasma-free using a mycoplasma detection kit (Thermo Fisher Scientific, AAJ66117AMJ) and were maintained at 37°C and 5% CO_2_.

### Cell proliferation assay.

The colony formation assay was performed to evaluate GBM cell proliferation as we previously described (22). GBM cells were seeded into the 6-well plates at 1,000 cells per well for overnight incubation. GBM cells were then pretreated with cilengitide, LY294002, or SC75741 for 1 hour. LGMN recombinant protein was then added to the corresponding wells. After 24 hours of the treatment, GBM cells were continuously cultured with fresh DMEM containing 10% FBS for 12 days. At the end of the incubation, GBM cells were stained with 0.25% crystal violet. The colony numbers were counted by ImageJ (NIH). The dose-response curves and IC_50_ were determined using GraphPad Prism. Moreover, we used the Incucyte Live Cell Analysis System (Sartorius) to monitor GBM cell proliferation. The time-lapse images were captured every 2 hours. The proliferation rate was calculated as the confluence of GBM cells at each time point subtracted by the confluence of the GBM cells at 0 hours. To assess the proliferation of Raw264.7 or THP1 macrophages, wells were stained with the CellTrace Violet Cell Proliferation Kit (Invitrogen, C34557) for 20 minutes at 37°C. Cells were then cultured in the dark with different treatments for 3 days before being subjected to flow cytometry analysis. The percentage of CellTrace Violet–positive peaks compared with the undivided peak (generation 0) was analyzed using ImageJ software.

### Apoptosis analysis.

GBM cell apoptosis was determined using Apotracker Green (BioLegend, 427402) as described previously (24). Briefly, GBM cells were harvested and stained with Apotracker (1:10 dilution) after the treatment. Cells were incubated with propidium iodide (PI) solution (BioLegend, 421301) for labeling of late apoptotic and necrotic GBM cells. PI and fluorescein isothiocyanate (FITC) signals were recorded and analyzed in a BD FACSymphony flow cytometer. The result was further analyzed using FlowJo v10.8.1.

### Mice and intracranial xenograft tumor models.

Female C57BL/6 and nude mice were purchased from The Jackson Laboratory (0000664 and 007850, respectively). The LGMN-mKD mice were generated by bone marrow transplantation. In brief, recipient mice received 1,100 cGy total-body radiation with an XRad320 Irradiator (Precision X-Ray). After 24 hours, recipient mice were injected with donor-derived shRNA control (shC) or *Lgmn* shRNA (sh*Lgmn*) bone marrow cells intravenously. The orthotopic intracranial xenograft GBM mouse models were established as we described previously (24, 37, 95). For the coinjection model, mouse Raw264.7 macrophages were incubated with the CM collected from CT2A cells for 24 hours. The CT2A CM–educated Raw264.7 macrophages were then mixed with CT2A cells at a 1:1 ratio and injected into the brains of C57BL/6 mice. According to the IACUC protocol, we sacrificed any mice exhibiting neurological deficits or moribund appearance during the treatment. To obtain tumor samples for IF analysis, mouse brains were isolated by transcardiac perfusion of PBS and 4% paraformaldehyde (PFA) to preserve the cellular architecture of GBM tumors. The isolated brains were preserved in 4% PFA until processing for cryosectioning.

### Cryosectioning and IF staining.

The mouse brain was transferred from 4% PFA to a 50 mL Falcon tube with 15% sucrose in PBS containing 0.01% sodium azide for 48 hours. Then the brains were further preserved in 30% sucrose for another 48 hours. OCT compound was used to embed the entire brain fully. Then the OCT-embedded samples were frozen at –80°C and ready for cryosectioning. A Leica CM1860 UV Cryostat was used to slice the brain into 10 μm sections. IF analysis was conducted using an established protocol as we previously described (37, 95). Primary antibodies against the following proteins were used: F4/80 (Cell Signaling Technology [CST], 70076), CD69 (Santa Cruz Biotechnology, sc-373799), Ki67 (CST, 9129), or cleaved caspase-3 (CST, 9661). For costaining of CD69 with CD8 and CD4, GBM tumor slides were incubated with the secondary antibody against CD69 with either FITC-conjugated CD4 antibody (CST, 96127) or FITC-conjugated CD8 antibody (CST, 35467) for 1 hour at room temperature. After washing with PBS, cell nuclei were counterstained with SlowFade Gold Antifade Mountant with DAPI (Invitrogen, S36938). ImageJ assessed the relative level of the target protein signal intensity.

### Isolation of mouse primary BMDMs.

The primary bone marrow–derived macrophages (BMDMs) were isolated from healthy female C57BL/6 mice and cultured as we described previously (37). The phenotype of BMDMs was confirmed by flow cytometry analysis.

### Computational and scRNA-Seq analysis of human GBM datasets.

TCGA-GBM microarray gene expression dataset (Agilent, 4502A) and the CGGA GBM dataset from GlioVis (http://gliovis.bioinfo.cnio.es/) were downloaded for calculation of the correlation between *ITGAV* and *LGMN* expressions and GSEA. The procedures of gene correlation analysis and GSEA of gene signatures of interest were conducted as we reported previously (22–24, 37). The *ITGAV* expression in different cell populations of GBM tumor tissues was analyzed using data from the Brain TIME dataset (96). All the scRNA-Seq analyses were conducted in the Talk2Data platform (BioTuring). scRNA-Seq data from the European Genome-Phenome Archive (EGAS00001004422) were used to identify *LGMN*-high and -low TAM subclusters in GBM tumor tissues (97). To identify downstream targets of macrophage-derived LGMN in GBM cells, scRNA-Seq data from the Gene Expression Omnibus (GEO) database (GSE182109) and EGAS00001004422 were used to classify GBM patients into *LGMN*-high and -low TAM groups (29, 97). The scRNA-Seq data of GBM cells from these 2 groups were downloaded from each study for GSEA for the human hallmark gene set. We overlapped the top 10 pathways from each screen to determine potential pathway candidates.

### Plasmids and viral transfections.

shRNAs targeting mouse *Lgmn*, human *LGMN*, mouse *Itgav*, and human *ITGAV* in the pLKO.1 vector (Sigma-Aldrich, SHC001) were used in this study. 293T cells were transfected with the mixture of packaging vectors psPAX2 (4 mg; Addgene, 12260) and pMD2.G (2 mg; Addgene, 12259) and target vector for generating lentiviral particles (8 mg) as we previously described (24, 37, 77, 95). After 48 hours of transfection, CM containing lentiviral particles was collected from 293T cells. Macrophages or GBM cells were treated with viral supernatant and 10 μg/mL Polybrene (Millipore, TR-1003-G). After 48 hours of transfection, 2 mg/mL puromycin (Millipore, 540411) was used to select successfully transfected cells. The following shRNA sequence was selected for further functional validation: *Lgmn*: #4, TRCN0000029256, and #5, TRCN0000276301; *LGMN*: #11, TRCN0000029256, and #12, TRCN0000029258; *ITGAV*: #11, TRCN0000003239, and #12, TRCN0000003240; *Itgav*: #9, TRCN0000066590, and 12, TRCN0000066591.

### Immunoblotting.

The protein expression level in macrophages and GBM cells was determined by Western blotting analysis using an established protocol as we described previously (37, 42, 95). Primary antibodies against the following proteins were used: LGMN (CST, 93627S), p-AKT (CST, 3787), AKT (CST, 4691), GSK3β (CST, 12456S), p-GSK3β (Invitrogen, 44-604G), ERα (Invitrogen, PA1-309), ERβ (Invitrogen, MA5-24807), PAX3 (Calbiochem, CA1010), p-STAT3 (CST, 9145), STAT3 (CST, 9139), integrin α_v_ (CST, 4711), p-p65 (CST, 3033), p65 (CST, 6956), p-ERK (CST, 4370), ERK (CST, 4695), actin (CST, 3700), and PD-L1 (CST, 64988). HRP-linked anti-mouse (CST, 7076) or anti-rabbit (CST, 7074) secondary antibodies were used accordingly. Target protein signals were captured by the ChemiDoc Imaging System (Bio-Rad).

### Immune cell isolation and flow cytometry.

Tumor-infiltrated macrophages were extracted using the Percoll density gradient cell separation method as we previously described (24, 77). For spleen cell isolation, tissues were homogenized on ice with pre-cold RPMI containing 2% FBS. To remove red blood cells, we incubated the samples with ACK buffer (Thermo Fisher Scientific, A1049201) for 10 minutes on ice. After washing with RPMI containing 10% FBS, samples were centrifuged at 300*g* for 10 minutes at 4°C. The single-cell suspensions were incubated with fixable viability dye (Invitrogen, 5211229035) on ice for 10 minutes to label dead cells, and incubated with True-Stain Monocyte Blocker (BioLegend, 426102) and TruStain FcX (anti–mouse CD16/32) antibody (BioLegend, 103132) for 30 minutes on ice to block nonspecific binding sites and Fc receptors. For macrophage staining, an antibody cocktail containing PerCP/Cy5.5–anti–mouse CD45 (BioLegend, 103132), PE/Cy7–anti–mouse/human CD11b (BioLegend, 101216), Alexa Fluor 700–anti–mouse Ly6C (BioLegend, 128024), FITC–anti–mouse Ly6G (BioLegend, 127606), and Pacific blue–anti–mouse F4/80 (BioLegend, 123124) was added to single-cell suspensions. Another antibody cocktail containing PerCP/Cy5.5–anti–mouse CD45 and PE/Cy7–anti–mouse/human CD11b was prepared separately and used before intracellular staining of CD68. Cells were fixed in fixation buffer at room temperature for 20 minutes. After washing in permeabilization buffer twice, cells were incubated with PE–anti–mouse CD68 (BD Biosciences, 566386) for 20 minutes at room temperature. For staining with p-AKT and p-p65, cells were fixed in a fixation buffer at room temperature for 15 minutes and permeabilized in 90% cold methanol for 15 minutes on ice following a previously described protocol (24). Isolated cells were resuspended with p-AKT or p-p65 primary antibody overnight at 4°C, and then incubated with goat anti-rabbit IgG cross-adsorbed secondary antibody (AF594, CST, 8889S). After rewashing, cells were read in a BD FACSymphony flow cytometer.

For T cell staining, an antibody cocktail containing BUV395–anti–mouse CD4 (BD Biosciences, 740208), Percp/Cy5.5–anti–mouse CD45 (BioLegend, 103132), AF488–anti–mouse CD3 (BioLegend, 100210), PE/Cy7–anti–mouse CD69 (BioLegend, 104512), and BV711 anti–mouse CD8 (BioLegend, 100747) was added to single-cell suspensions of splenic cells. After 1 hour of incubation on ice, cells were pelleted by centrifuging at 300*g* for 5 minutes and resuspended in the fixation buffer (BioLegend, 420801) overnight at 4°C. A BD FACSymphony or BD LSRFortessa flow cytometer was used to analyze IF intensity of cells. The result was further analyzed using FlowJo v10.8.1.

### Migration assay.

Transwell migration assay was performed on human and mouse macrophages as described previously (77, 95). After the treatment, macrophages were incubated with serum-free medium and seeded into 5.0 mm permeable polycarbonate membrane inserts (Corning, 07-200-149). Human LGMN recombinant protein (OriGene, TP324975) or mouse LGMN recombinant protein (OriGene, TP727290) with serum-free medium was added to the receiver wells. The migrated macrophages were fixed with 10% PFA after 10 hours of incubation in the Transwell plate. Migrated cells were stained with crystal violet (Sigma-Aldrich, C-3886) and captured under an EVOS microscope (Thermo Fisher Scientific). ImageJ (NIH) was used to count the migrated macrophages. The Incucyte Live Cell Analysis System was used to record the trajectories of macrophages as we previously described (24). Briefly, macrophages were seeded into 96-well plates and treated with inhibitor and LGMN recombinant protein. The plate was then cultured in the Incucyte for 24 hours. A series of images was captured every 15 minutes. TrackMate was used to reconstruct and analyze the time-lapse images (47, 48). The speed of macrophage movement was calculated for each reconstructed track.

### Statistics.

Statistical analysis in this study was conducted using GraphPad Prism 10. Comparison between 2 groups was conducted using 2-tailed Student’s *t* test, and comparisons among multiple groups were performed using a 1-way ANOVA test in Tukey’s method. For the Incucyte-recorded proliferation assay, a 2-way ANOVA test was used to determine the statistical difference between each proliferation curve. Pearson’s test was used to determine the *P* value and *R* value. The survival analysis for GBM mouse models was conducted with the log-rank (Mantel-Cox) test. *P* values of less than 0.05 were considered significant. The data are presented as the means ± SEM.

### Study approval.

All animal protocols were approved by the Northwestern University and Cleveland Clinic Institutional Animal Care and Use Committees.

### Data availability.

The data supporting the findings of this study are available within this article and within the Supporting Data Values file. External single-cell data used in this study are available in the NCBI’s GEO database (GSE182109) and the European Genome-Phenome Archive (EGAS00001004422).

## Author contributions

LP, SG, YH, FK, YL, and FZ performed experiments. PC conceived the project. LP, SG, and YH acquired data. LP, SG, and JDL analyzed and interpreted data. LP and PC wrote the manuscript, and all authors participated in editing the paper. The final manuscript was approved by all authors.

## Supplementary Material

Supplemental data

Unedited blot and gel images

Supplemental video 1

Supplemental video 2

Supplemental video 3

Supplemental video 4

Supplemental video 5

Supplemental video 6

Supplemental video 7

Supplemental video 8

Supplemental video 9

Supplemental video 10

Supplemental video 11

Supplemental video 12

Supplemental video 13

Supplemental video 14

Supplemental video 15

Supplemental video 16

Supplemental video 17

Supplemental video 18

Supplemental video 19

Supplemental video 20

Supplemental video 21

Supplemental video 22

Supplemental video 23

Supporting data values

## Version 1 03/25/2025

In-Press Preview

## Version 2 05/15/2025

Electronic publication
