# Supplementary material for: Targeting legumain-mediated cell-cell interaction sensitizes glioblastoma to immunotherapy in preclinical models
Source: J Clin Invest. 2025 Mar 25;135(10):e186034. doi: 10.1172/JCI186034 (PMC12077903; doi:10.1172/JCI186034)
Supplement: Supplemental data [file jci-135-186034-s058.pdf]

Supplemental Figure 1

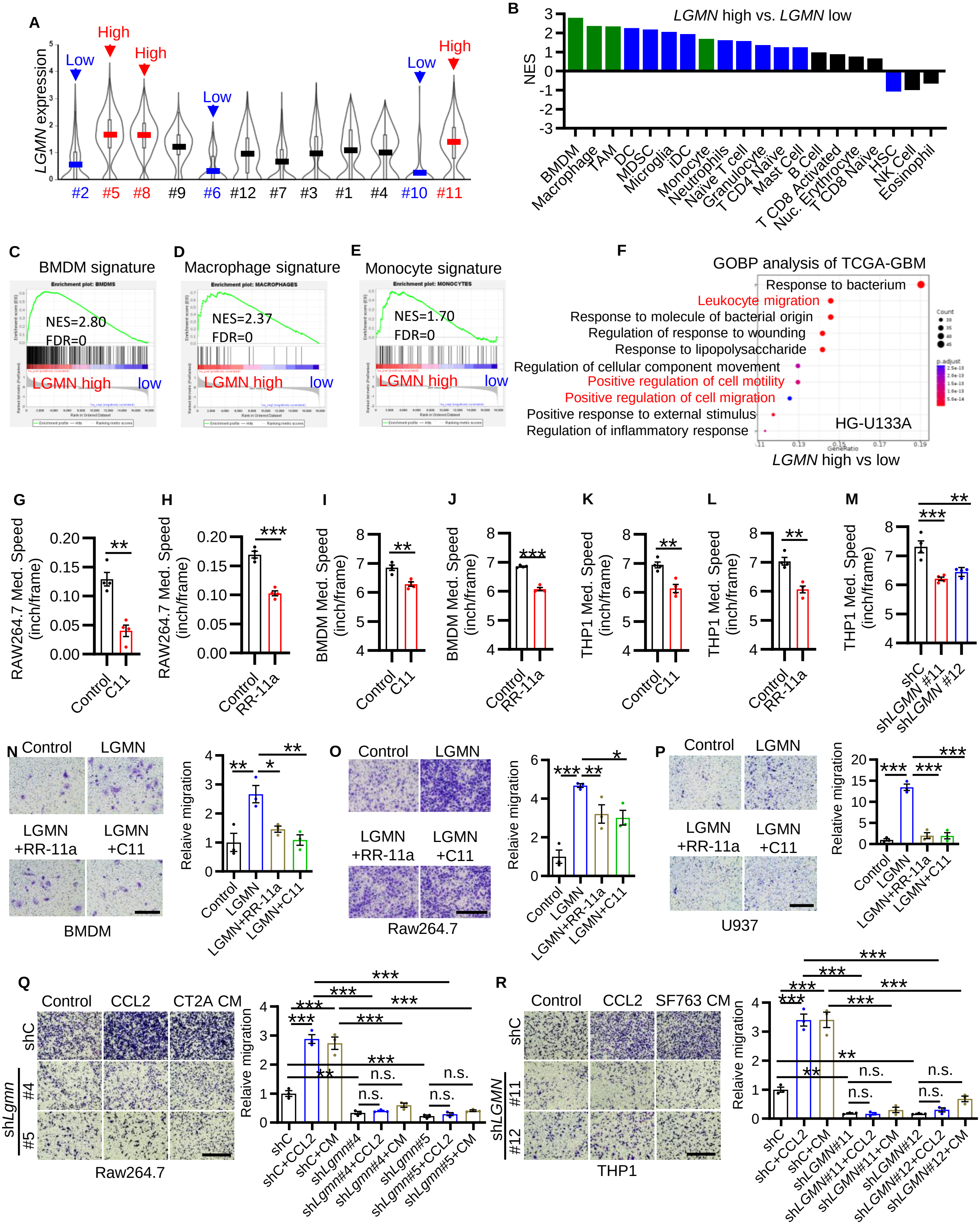

Supplemental Figure 1. Inhibition of LGMN suppresses macrophage migration. (**A**) *LGMN* expression in different macrophage subclusters was profiled by analyzing scRNA-seq data (EGAS00001004422). The mean expression values are indicated. (**B**) Gene Set Enrichment Analysis (GSEA) showing various types of immune cells in *LGMN* high compared to *LGMN* low tumors of the TCGA GBM dataset. BMDMs and macrophages are the most enriched immune cells in *LGMN* high group. Top and bottom quartiles were set as high and low, respectively. Blue bars indicate FDR < 0.25. Green bars indicate the signatures related to macrophages and monocytes. (**C-E**) GSEA shows the enrichment of bone marrow-derived macrophage (BMDM) signature (**C**), macrophage signature (**D**), and monocyte signature (**E**) in *LGMN* high compared with *LGMN* low TCGA patient tumors. The NES and FDR q values are shown. (**F**) Gene Ontology Biological Process (GOBP) analysis shows the pathways enriched in the *LGMN* high TCGA GBM tumors. (**G** and **H**) Quantification of the movement speed of Raw264.7 macrophages treated with C11 (1 μmol/L, **G**) (see Supplemental Video 1 and 2) or RR-11a (20 nmol/L, **H**) (see Supplemental Video 3 and 4). n = 4 independent samples. (**I** and **J**) Quantification of the movement speed of BMDMs treated with C11 (1 μmol/L, **I**) (see Supplemental Video 5 and 6) or RR-11a (20 nmol/L, **J**) (see Supplemental Video 7 and 8). n = 4 independent samples. (**K** and **L**) Quantification of the movement speed of THP1 macrophages treated with C11 (1 μmol/L, **K**) (see Supplemental Video 9 and 10) or RR-11a (20 nmol/L, **L**) (see Supplemental Video 11 and 12). (**M**) Quantification of the movement speed of human THP1 macrophages expressing shRNA control (shC) and *LGMN* shRNAs (sh*LGMN*) (see Supplemental Video 13-15). n = 4 independent samples. (**N-P**) Representative and quantification of relative migration of BMDMs (**N**), Raw264.7 macrophages (**O**), and U937 macrophages (**P**) following treatment of LGMN recombinant protein (10 ng/mL) with or without RR-11a (20 nmol/L) or C11 (1 μmol/L). Scale bars, 200 μm. n = 3 independent samples. (**Q**) Representative and quantification of relative migration of Raw264.7 macrophages transfected with shC or sh*Lgmn* and treated with CCL2 (10 nmol/L) or CT2A cell conditioned media (CM). Scale bars, 200 μm. n = 3 independent samples. (**R**) Representative and quantification of relative migration of THP1 macrophages transfected with shC or sh*LGMN*, and treated with CCL2 (10 nmol/L) or SF763 cell CM. Scale bars, 200 μm. n = 3 independent samples. Data from multiple replicates are presented as mean ± SEM. *, *P* < 0.05, **, *P* < 0.01, ***, *P* < 0.001, Student's t test (G-M), One-way ANOVA test (N-R).

Supplemental Figure 2

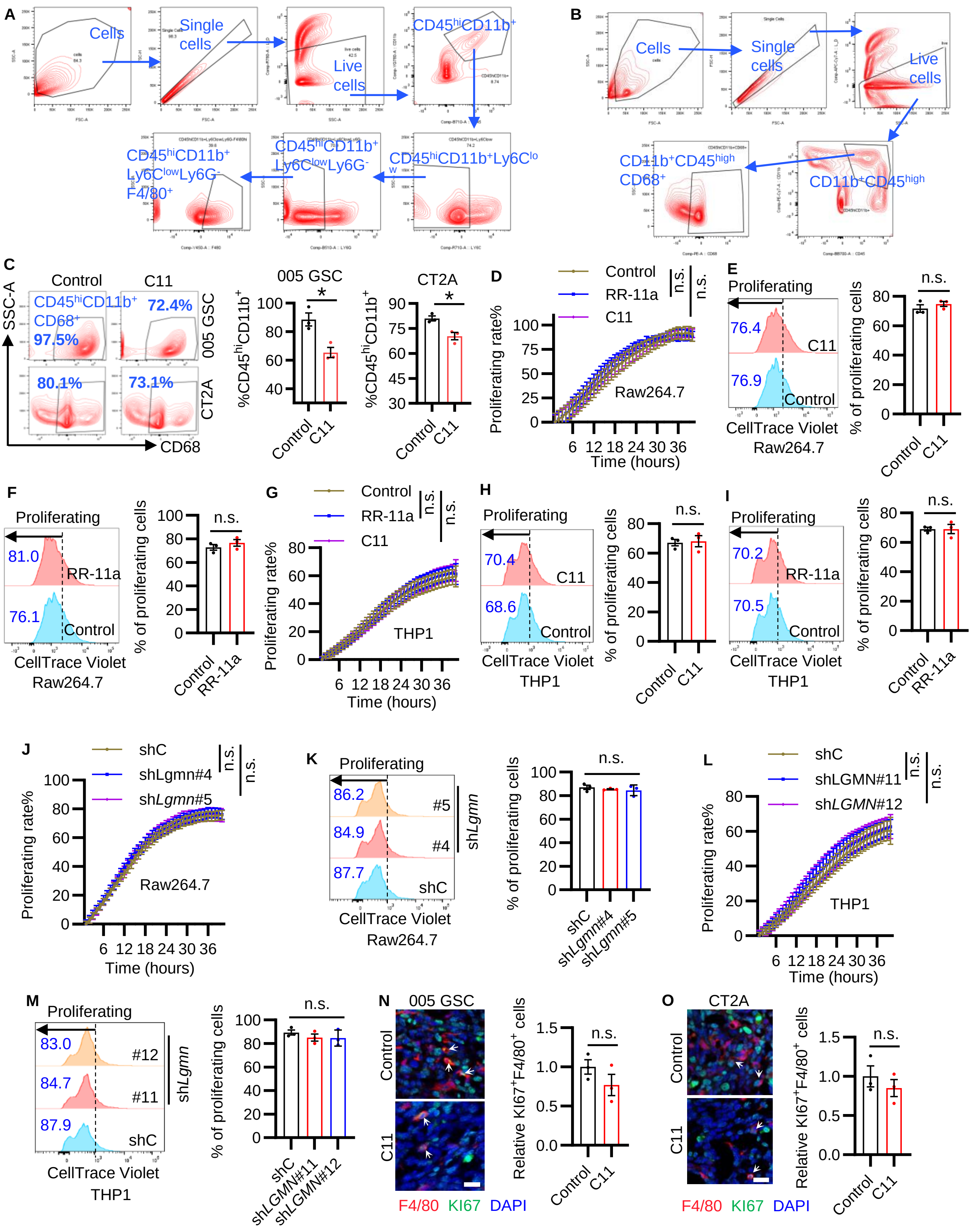

Supplemental Figure 2. Inhibition of LGMN reduces macrophage infiltration but does not affect macrophage proliferation. (**A** and **B**) Gating strategies for analyzing the percentage of tumor-infiltrating $CD45^{hi}CD11b^{+}Ly6C^{low}Ly6G^{-}F4/80^{+}$ macrophages (**A**) and $CD11b^{+}CD45^{hi}CD68^{+}$ macrophages (**B**) from GBM tumor-bearing mice. (**C**) Representative images and quantification of flow cytometry for the percentage of $CD45^{hi}CD11b^{+}CD68^{+}$ macrophages in size-matched control and C11-treated 005 GSC and CT2A tumors in C57BL/6 mice. C11 (10 mg/kg/day) was intraperitoneally (i.p.) administered in tumor-bearing mice. n = 3 independent samples. (**D**) Proliferation curves of Raw264.7 macrophages treated with RR-11a (20 nmol/L) or C11 (1 μmol/L). Macrophage proliferation was recorded and analyzed using the Incucyte imaging system. n = 6 independent samples. (**E** and **F**) Representative and quantification of proliferation in CellTrace Violet-labeled Raw264.7 macrophages treated with C11 (1 μmol/L) (**E**) or RR-11a (20 nmol/L) (**F**). The percentage of proliferating cells for each group is indicated. n = 3 independent samples. (**G**) Proliferation curves of THP1 macrophages treated with RR-11a (20 nmol/L) or C11 (1 μmol/L). Macrophage proliferation was recorded and analyzed using the Incucyte imaging system. n = 6 independent samples. (**H** and **I**) Representative and quantification of proliferation in CellTrace Violet-labeled THP1 macrophages treated with C11 (1 μmol/L) (**H**) or RR-11a (20 nmol/L) (**I**). The percentage of proliferating cells for each group is indicated. n = 3 independent samples. (**J**) Proliferation curves of Raw264.7 macrophages expressing shRNA control (shC) and *Lgmn* shRNA (sh*Lgmn*). Macrophage proliferation was recorded and analyzed using the Incucyte imaging system. n = 6 independent samples. (**K**) Representative and quantification of proliferation in CellTrace Violet-labeled Raw264.7 macrophages expressing shC and sh*Lgmn*. The percentage of proliferating cells for each group is indicated. n = 3 independent samples. (**L**) Proliferation curves of THP1 macrophages expressing shC and sh*LGMN*. Macrophage proliferation was recorded and analyzed using the Incucyte imaging system. n = 6 independent samples. (**M**) Representative and quantification of proliferation in CellTrace Violet-labeled THP1 macrophages expressing shC and sh*LGMN*. The percentage of proliferating cells for each group is indicated. n = 3 independent samples. (**N** and **O**) Immunofluorescence and quantification of relative $F4/80^{+}Ki67^{+}$ proliferating macrophages in tumors from the 005 GSC (**N**) and CT2A (**O**) mouse models treated with or without C11 (10 mg/kg, i.p., daily). Scale bar, 25 μm. n = 3 independent samples. Data from multiple replicates are presented as mean ± SEM. *, $P < 0.05$, **, $P < 0.01$, n.s., not significant, Student's t test (C, E, F, H, I, N, and O), One-way ANOVA test (K and M), Two-way ANOVA test (D, G, J, and L).

Supplemental Figure 3

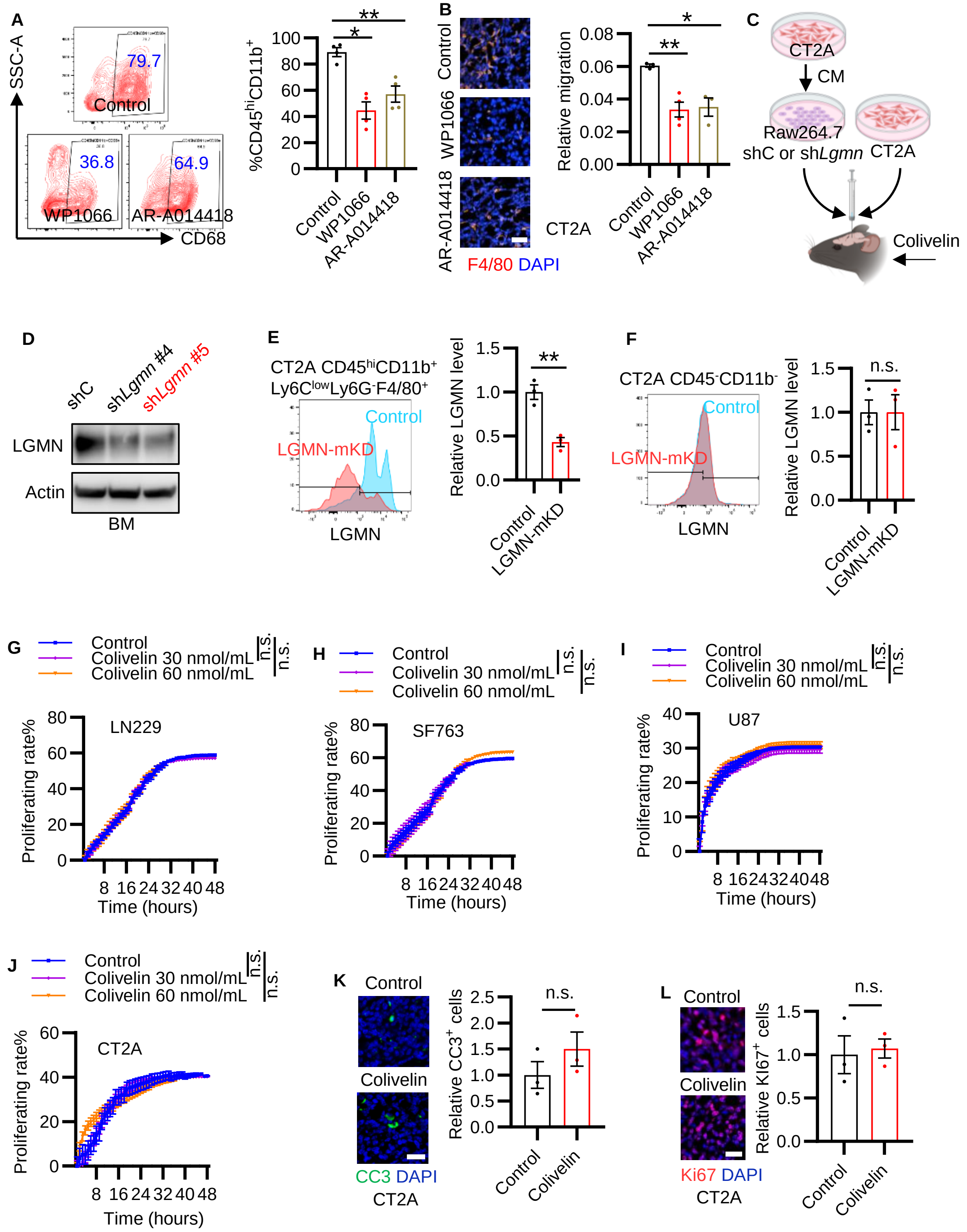

Supplemental Figure 3. Inhibition of the GSK3β-STAT3 axis reduces macrophage infiltration and tumor progression. (**A**) Representative images and quantification of flow cytometry for the percentage of $CD45^{hi}CD11b^{+}CD68^{+}$ macrophages in size-matched WP1066 and AR-A014418-treated CT2A tumors from C57BL/6 mice. WP1066 (30 mg/kg/day, i.p.) and AR-A014418 (30 mg/kg/day, i.p.) were administered in tumor-bearing mice. n = 4 independent samples. (**B**) Representative images and quantification of immunofluorescence for $F4/80^{+}$ macrophages in size-matched WP1066 (30 mg/kg/day, i.p.) and AR-A014418 (30 mg/kg/day, i.p.)-treated CT2A tumors from C57BL/6 mice. n = 4 independent samples. (**C**) Diagram showing the procedures of co-injection of CT2A cells and CT2A CM-educated Raw264.7 macrophages expressing shRNA control (shC) and *Lgmn* shRNA (sh*Lgmn*) into the brains of C57BL/6 mice. Mice were treated with or without Colivelin (30 mg/kg body weight, i.p., every other day). (**D**) Immunoblots for LGMN in lysates of bone marrow (BM) cells expressing shC and sh*Lgmn*. (**E** and **F**) Representative and quantification of flow cytometry for LGMN expression in tumor-infiltrating $CD45^{hi}CD11b^{+}Ly6C^{low}Ly6G^{-}F4/80^{+}$ macrophages (**E**) and $CD45^{-}CD11b^{-}$ cancer cells (**F**) isolated from CT2A tumor-bearing control or LGMN macrophage-specific knockdown (LGMN-mKD) mice. (**G-J**) Proliferation curves of LN229 (**G**), SF763 (**H**), U87 (**I**), and CT2A (**J**) cells treated with Colivelin at indicated concentrations. GBM cell proliferation was recorded and analyzed using the Incucyte imaging system for 48 hrs. n = 6 independent samples. (**K** and **L**) Immunofluorescence and quantification of relative CC3 (**K**) and Ki67 (**L**) expression in tumors from the CT2A mouse models treated with or without Colivelin (30 mg/kg body weight, i.p., every other day). Scale bar, 25 μm. n = 3 independent samples. Data from multiple replicates are presented as mean ± SEM. *, $P < 0.05$, **, $P < 0.01$, n.s., not significant, Student's t test (E, F, K, and L), One-way ANOVA test (A and B), Two-way ANOVA test (G-J).

Supplemental Figure 4

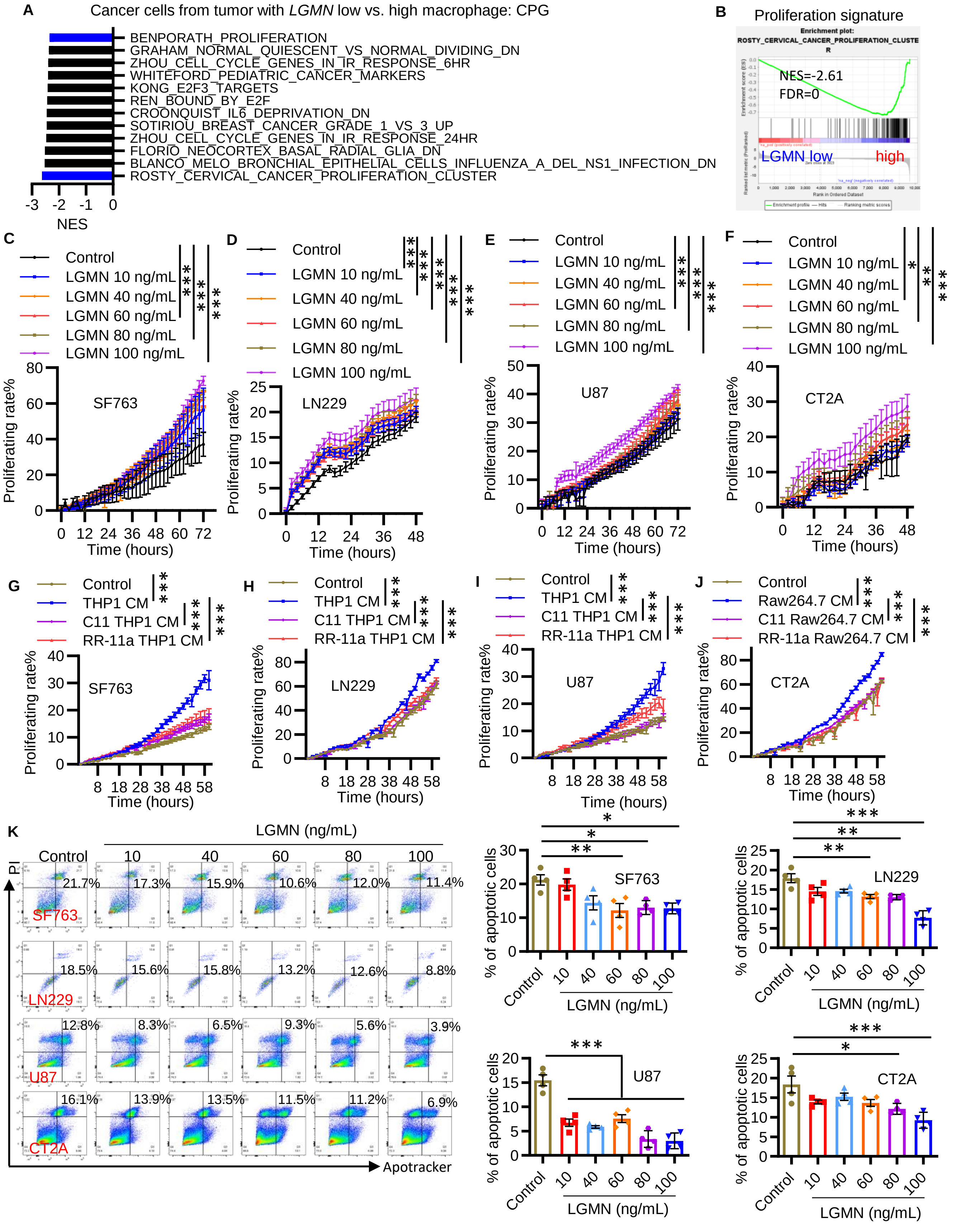

Supplemental Figure 4. LGMN regulates GBM cell proliferation and apoptosis. (**A**) Gene Set Enrichment Analysis (GSEA) shows the top Chemical and Genetic Pertubations (CGP) pathways in cancer cells of GBM tumors containing LGMN low versus high macrophage LMGN based on the scRNA-seq dataset (EGAS00001004422). NES, normalized enrichment score. (**B**) GSEA shows the enrichment of cancer proliferation cluster signature in the macrophage *LGMN* low group compared with macrophage *LGMN* high group. NES and false discovery rate (FDR) q values are shown. (**C-F**) Proliferation curves of SF763 (**C**), LN229 (**D**), U87 (**E**), and CT2A (**F**) cells treated with LGMN recombinant protein at indicated concentrations. GBM cell proliferation was recorded and analyzed using the Incucyte imaging system for 48 hrs. n = 6 independent samples. (**G-J**) Proliferation curves of SF763 (**G**), LN229 (**H**), U87 (**I**), and CT2A (**J**) cells treated with the conditioned media (CM) from THP1 or Raw264.7 macrophages, which were pretreated with C11 (1 μmol/L) or RR-11a (20 nmol/L). GBM cell proliferation was recorded and analyzed using the Incucyte imaging system for 60 hrs. n = 6 independent samples. (**K**) Representative images and quantification of Apotracker and PI staining showing the apoptosis of SF763, LN229, U87, and CT2A cells treated with LGMN recombinant protein at indicated concentrations. n = 4 independent samples. Data from multiple replicates are presented as mean $\pm$ SEM. *, $P < 0.05$, **, $P < 0.01$, ***, $P < 0.001$, Two-way ANOVA test (C-J), One-way ANOVA test (K).

Supplemental Figure 5

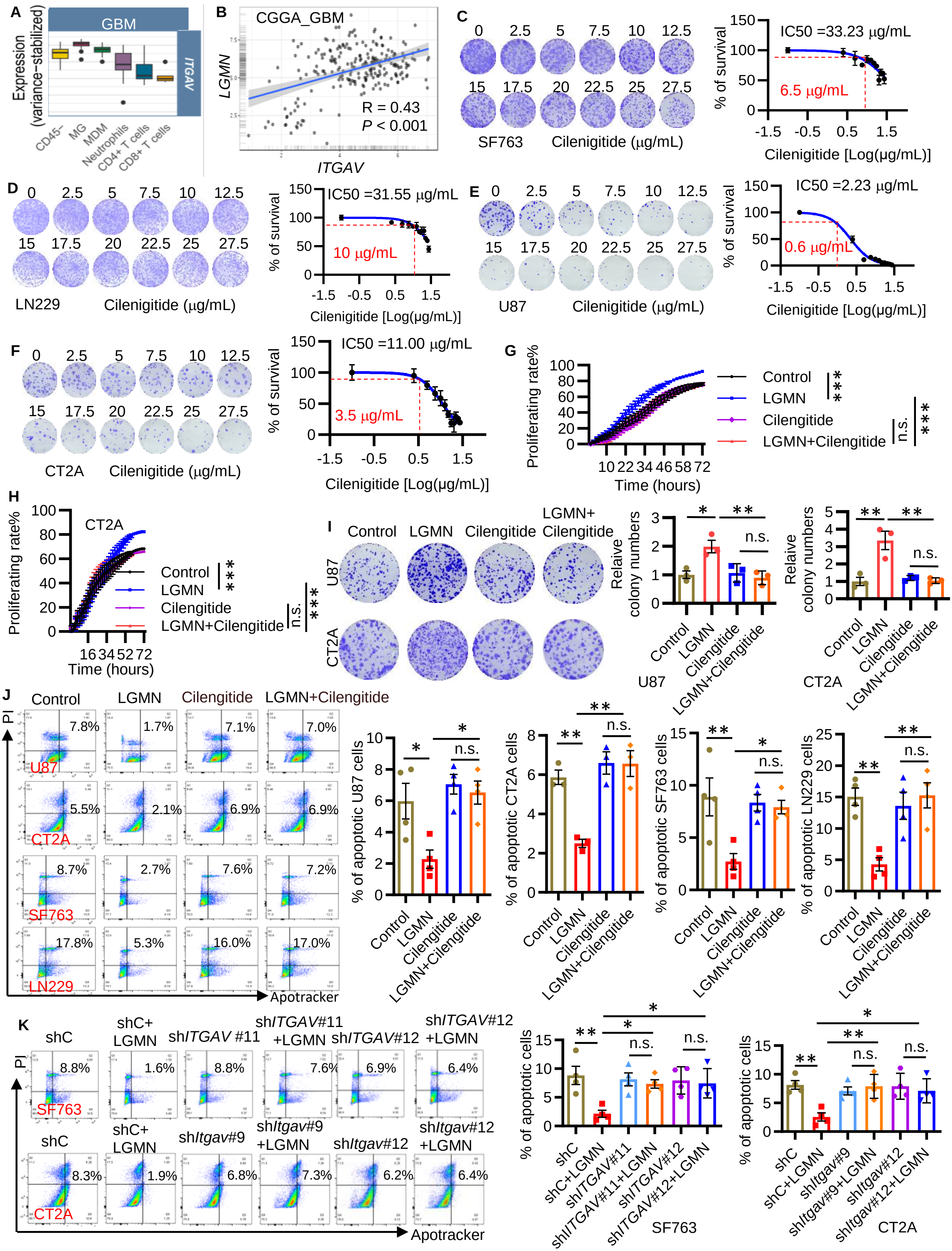

Supplemental Figure 5. Inhibition of integrin αV suppresses the regulatory role of LGMN in GBM cell proliferation and apoptosis. (**A**) *ITGAV* expression in distinct cell populations, including $CD45^-$ cancer cells, microglia (MG), macrophages (MDM), neutrophils, $CD4^+$ and $CD8^+$ T cells, of human GBM tumors from the Brain Tumor Immune Micro Environment (TIME) dataset. n = 6-15 independent samples. (**B**) Correlation analysis of *LGMN* and *ITGAV* expression in CGGA GBM dataset. R and *P* values are shown. (**C-F**) Representative images and dose-response curves of the colony formation assay showing the proliferation of SF763 (**C**), LN229 (**D**), U87 (**E**), and CT2A (**F**) cells incubated with Cilengitide at different concentrations. The IC50 and optimized concentration are indicated. n = 3 independent samples. (**G** and **H**) Proliferation curves of U87 (**G**) and CT2A (**H**) cells incubated with LGMN recombinant protein (100 ng/mL) in the presence or absence of integrin αV inhibitor Cilengitide (0.6 μg/mL for U87 and 3.5 μg/mL for CT2A). GBM cell proliferation was recorded and analyzed using the Incucyte imaging system for 72 hrs. n = 6 independent samples. (**I**) Representative images and quantification of the colony formation assay showing the proliferation of U87 and CT2A cells incubated with LGMN recombinant protein (100 ng/mL) in the presence or absence of Cilengitide (0.6 μg/mL for U87 and 3.5 μg/mL for CT2A). n = 3 independent samples. (**J**) Representative images and quantification of Apotracker and PI staining showing the apoptosis of U87, CT2A, SF763, and LN229 cells incubated with LGMN recombinant protein (100 ng/mL) in the presence or absence of Cilengitide (0.6 μg/mL for U87, 3.5 μg/mL for CT2A, 6.5 μg/mL for SF763, and 10 μg/mL for LN229). n = 3 independent samples. (**K**) Representative images and quantification of Apotracker and PI staining showing the apoptosis of SF763 and CT2A cells expressing shRNA control (shC) and *ITGAV* shRNA (sh*ITGAV*) and incubated with or without LGMN recombinant protein (100 ng/mL). n = 3 independent samples. Data from multiple replicates are presented as mean $\pm$ SD (A) or SEM (C-K). *, $P < 0.05$, **, $P < 0.01$, ***, $P < 0.001$, n.s., not significant, Two-way ANOVA test (G and H), One-way ANOVA test (I-K).

Supplemental Figure 6

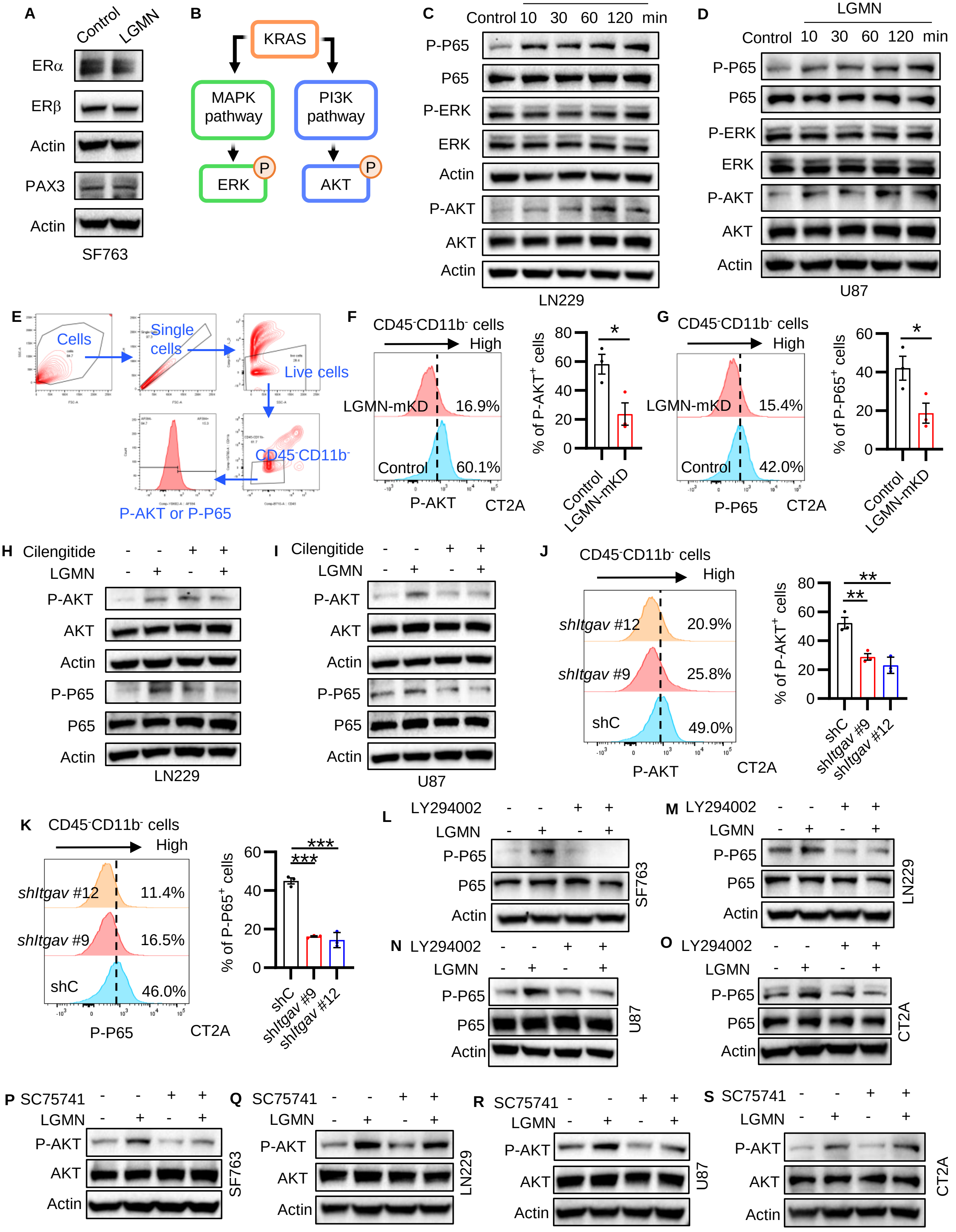

Supplemental Figure 6. Integrin αV-mediated LGMN regulates the AKT-P65 axis in GBM cells. (**A**) Immunoblots for ERα, ERβ, and PAX3 in cell lysates of SF763 cells treated with or without LGMN recombinant protein (100 ng/mL) for 24 hrs. (**B**) Diagram shows major downstream pathways of KRAS signaling. (**C** and **D**) Immunoblots for P-P65, P65, P-ERK, ERK, P-AKT, and AKT in cell lysates of LN229 (**C**) and U87 (**D**) cells treated with or without LGMN recombinant protein (100 ng/mL) for indicated time. (**E**) Gating strategy for P-AKT$^+$ and P-P65$^+$ GBM cells (CD45$^-$CD11b$^-$) from tumor tissues of CT2A GBM mouse model. (**F** and **G**) Representative and quantification of flow cytometry for P-AKT (**F**) and P-P65 (**G**) expression in CD45$^-$CD11b$^-$ GBM cells isolated from CT2A tumor-bearing control or LGMN macrophage-specific knockdown (LGMN-mKD) mice. (**H** and **I**) Immunoblots for P-P65, P65, P-AKT, and AKT in cell lysates of LN229 (**H**) and U87 (**I**) cells treated with LGMN recombinant protein (100 ng/L) in the presence or absence of integrin αV inhibitor Cilengitide (25 μg/mL). (**J** and **K**) Representative and quantification of flow cytometry for P-AKT (**J**) and P-P65 (**K**) expression in CD45$^-$CD11b$^-$ GBM cells isolated from CT2A tumors expressing shRNA control (shC) or *Lgmn* shRNAs (sh*Itgav*). (**L-O**) Immunoblots for P-P65 and P65 in cell lysates of SF763 (**L**), LN229 (**M**), U87 (**N**), and CT2A (**O**) cells treated with LGMN recombinant protein (100 ng/mL) in the presence or absence of AKT inhibitor LY294002 (10 μmol/L). (**P-S**) Immunoblots for P-AKT and AKT in cell lysates of SF763 (**P**), LN229 (**Q**), U87 (**R**), and CT2A (**S**) cells treated with LGMN recombinant protein (100 ng/mL) in the presence or absence of P65 inhibitor SC75741 (5 μmol/L). Data from multiple replicates are presented as mean ± SEM. *, $P < 0.05$, **, $P < 0.01$, ***, $P < 0.001$, Student’s t test (F and G), One-way ANOVA test (J and K).

Supplemental Figure 7

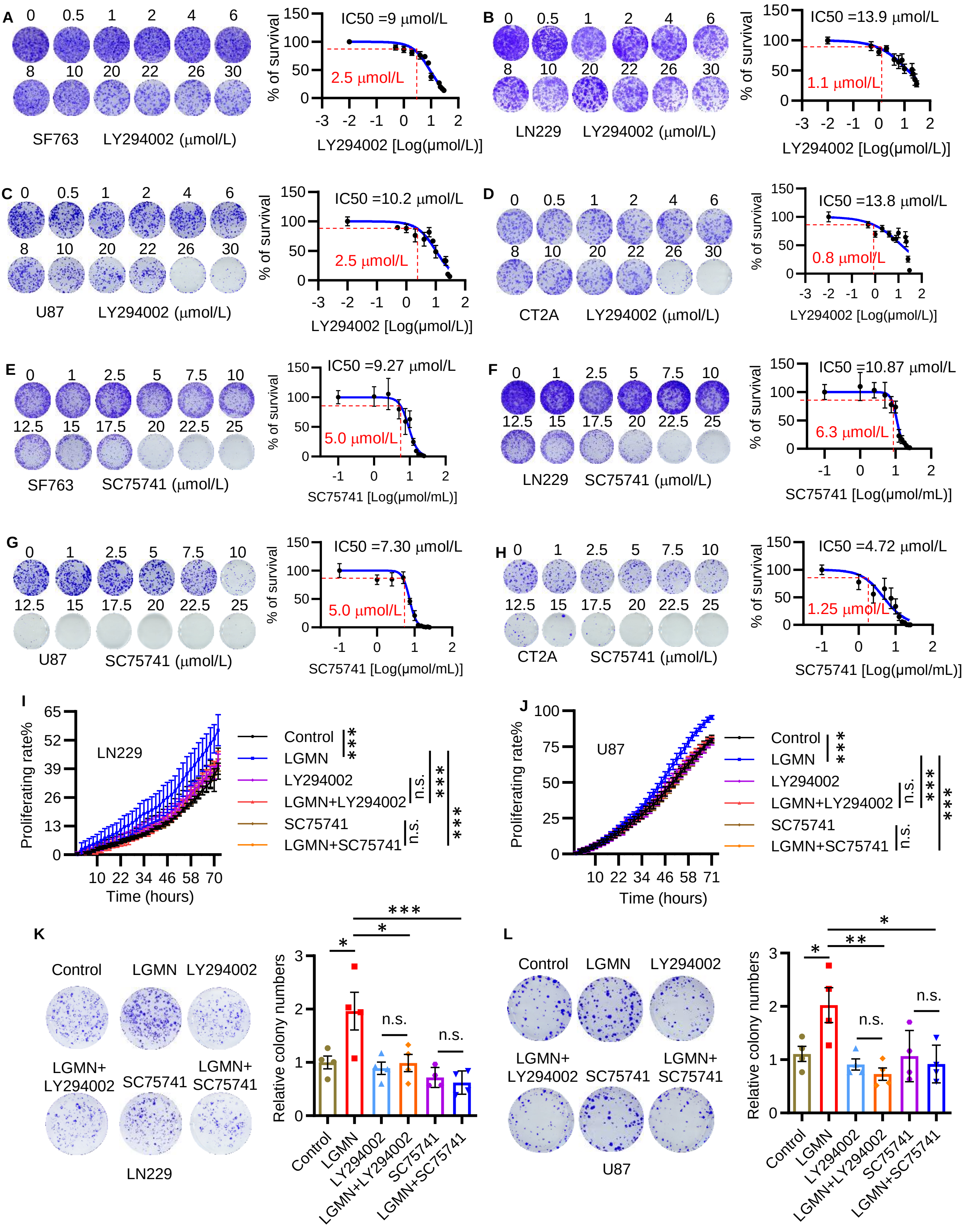

Supplemental Figure 7. LGMN-integrin αV-AKT-P65 axis promotes GBM cell proliferation. (**A-D**) Representative images and dose-response curves of the colony formation assay showing the proliferation of SF763 (**A**), LN229 (**B**), U87 (**C**), and CT2A (**D**) cells incubated with LY294002 at different concentrations. The IC50 and optimized concentration are indicated. n = 3 independent samples. (**E-H**) Representative images and dose-response curves of the colony formation assay showing the proliferation of SF763 (**E**), LN229 (**F**), U87 (**G**), and CT2A (**H**) cells incubated with SC75741 at different concentrations. The IC50 and optimized concentration are indicated. n = 3 independent samples. (**I** and **J**) Proliferation curves of LN229 (**I**) and U87 (**J**) cells incubated with LGMN recombinant protein (100 ng/mL) in the presence or absence of LY294002 (1.1 μmol/L for LN229 and 2.5 μmol/L for U87) or SC75741 (6.3 μmol/L for LN229 and 5 μmol/L for U87). n = 6 independent samples. (**K** and **L**) Representative images and quantification of the colony formation assay showing the proliferation of LN229 (**K**) and U87 (**L**) cells incubated with LGMN recombinant protein (100 ng/mL) in the presence or absence of LY294002 (1.1 μmol/L for LN229 and 2.5 μmol/L for U87) or SC75741 (6.3 μmol/L for LN229 and 5 μmol/L for U87). n = 4 independent samples. Data from multiple replicates are presented as mean ± SEM. *, $P < 0.05$, **, $P < 0.01$, ***, $P < 0.001$, n.s., not significant, Two-way ANOVA test (I and J), One-way ANOVA test (K and L).

Supplemental Figure 8

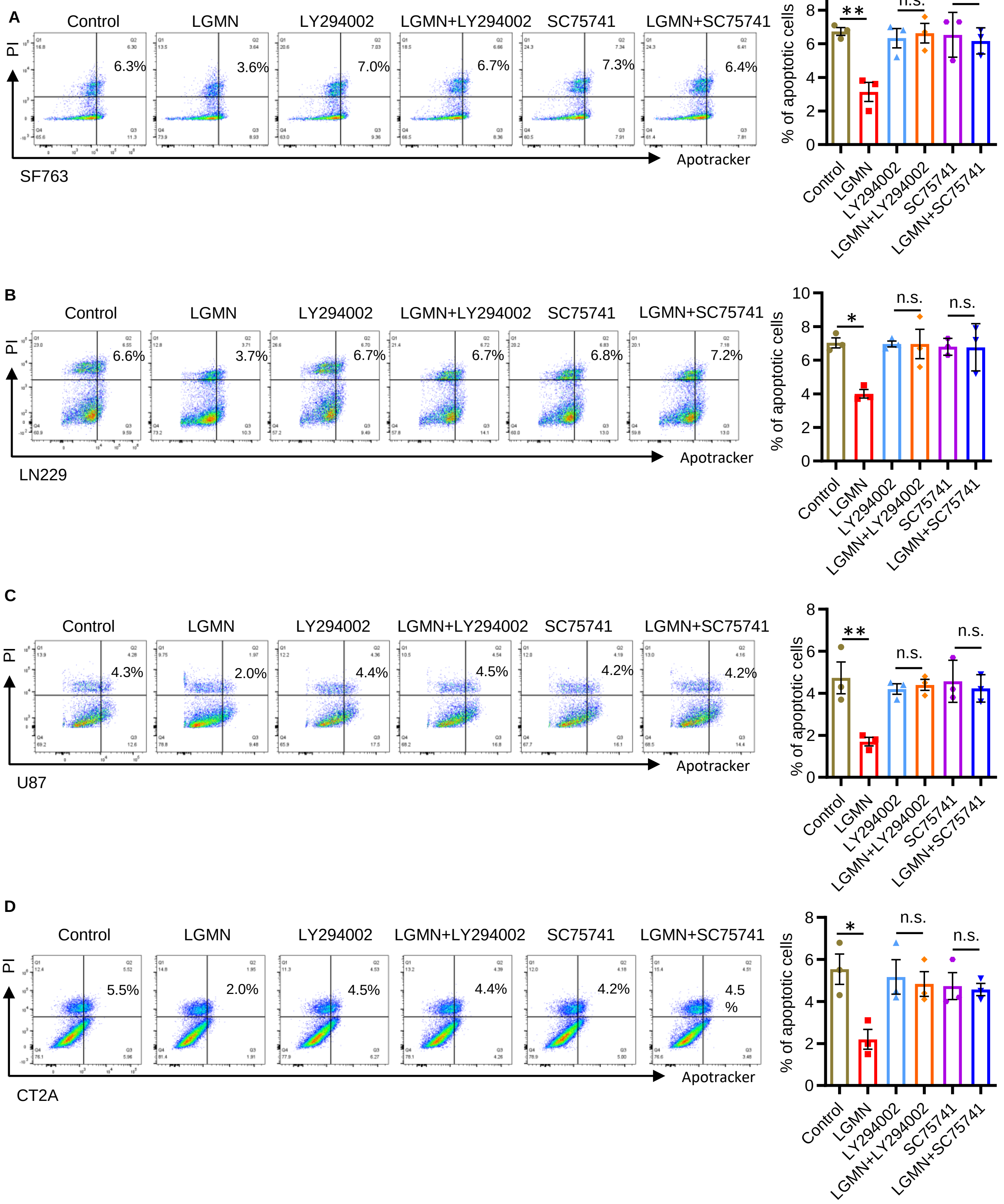

Supplemental Figure 8. LGMN inhibits GBM cell apoptosis by activating the AKT-P65 signaling axis. (**A-D**) Representative images and quantification of Apotracker and PI staining showing the apoptosis of SF763 (**A**), LN229 (**B**), U87 (**C**), and CT2A (**D**) cells incubated with LGMN recombinant protein (100 ng/L) in the presence or absence of AKT inhibitor LY294002 (2.5 μmol/L for SF763, 1.1 μmol/L for LN229, 2.5 μmol/L for U87, and 0.8 μmol/L for CT2A) or P65 inhibitor SC75741 (5 μmol/L for SF763, 6.3 μmol/L for LN229, 5 μmol/L for U87, and 1.25 μmol/L for CT2A). n = 3 independent samples. Data from multiple replicates are presented as mean ± SEM. *, $P < 0.05$, **, $P < 0.01$, n.s., not significant, One-way ANOVA test.

Supplemental Figure 9

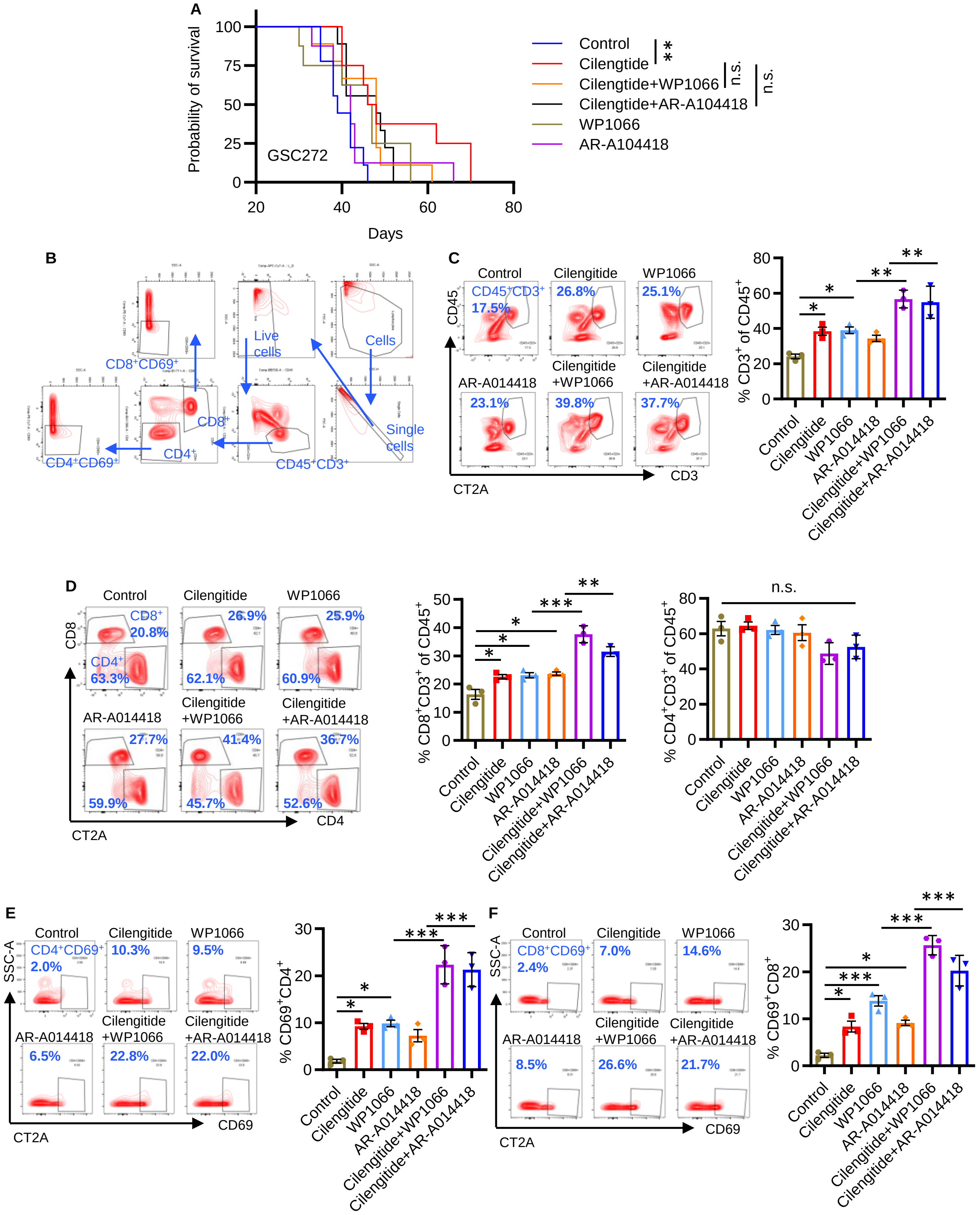

Supplemental Figure 9. Inhibition of GSK3β-STAT3 axis and integrin αV activates splenic T cells. (**A**) Survival curves of nude mice implanted with human GSC272 cells. Mice were treated with integrin αV inhibitor Cilengitide (30 mg/kg, i.p., daily), STAT3 inhibitor WP1066 (30 mg/kg, i.p., daily), and GSK3β inhibitor AR-A014418 (30 mg/kg, i.p., daily). n = 7 mice per group. (**B**) Gating strategy for analyzing T cells in spleen of GBM tumor-bearing mice. (**C**-**F**) Representative images and quantification of flow cytometry for the percentage of $CD45^+CD3^+$ cells (**C**), $CD45^+CD3^+CD4^+CD8^-$ cells, $CD45^+CD3^+CD8^+CD4^-$ cells (**D**), $CD45^+CD3^+CD4^+CD8^-CD69^+$ cells (**E**), and $CD45^+CD3^+CD8^+CD4^-CD69^+$ cells (**F**) in the spleen of size-matched CT2A tumors-bearing C57BL/6 mice. n = 3 independent samples. Data from multiple replicates are presented as mean ± SEM. *, $P < 0.05$, **, $P < 0.01$, ***, $P < 0.001$, n.s., not significant, One-way ANOVA test.

Supplemental Figure 10

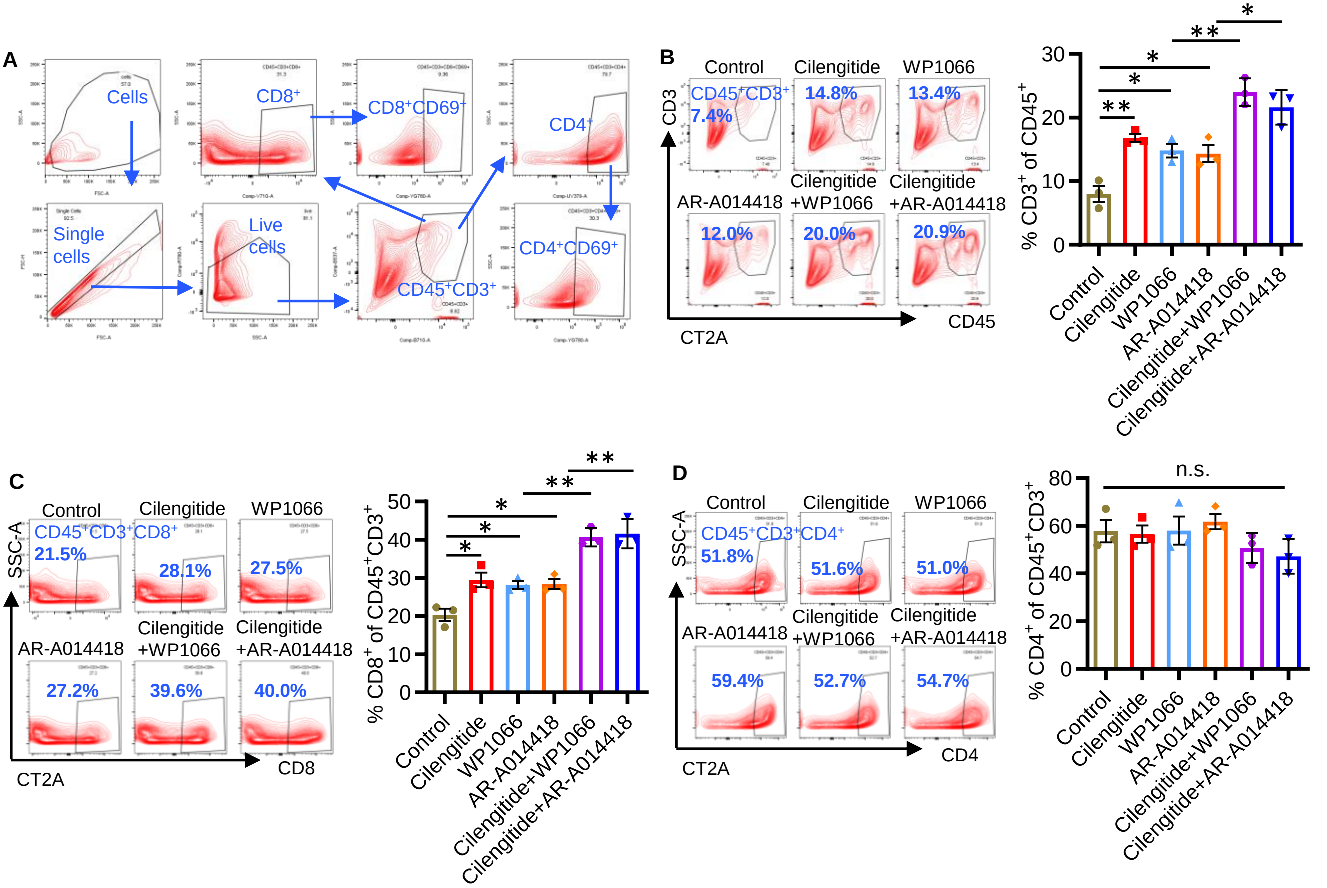

Supplemental Figure 10. Inhibition of GSK3β-STAT3 axis and integrin αV activates anti-tumor immunity. (**A**) Gating strategy for analyzing T cells in tumor tissues of CT2A GBM mouse model. (**B-D**) Representative images and quantification of flow cytometry for the percentage of $CD45^+CD3^+$ cells (**B**), $CD45^+CD3^+CD8^+$ cells (**C**), and $CD45^+CD3^+CD4^+$ cells (**D**) in the tumor tissues from size-matched CT2A tumors-bearing C57BL/6 mice. n = 3 independent samples. Data from multiple replicates are presented as mean ± SEM. *, $P < 0.05$, **, $P < 0.01$, ***, $P < 0.001$, n.s., not significant, One-way ANOVA test.
