# Supplementary material for: Targeting legumain-mediated cell-cell interaction sensitizes glioblastoma to immunotherapy in preclinical models
Source: J Clin Invest. 2025 Mar 25;135(10):e186034. doi: 10.1172/JCI186034 (PMC12077903; doi:10.1172/JCI186034)

Fig. 1L

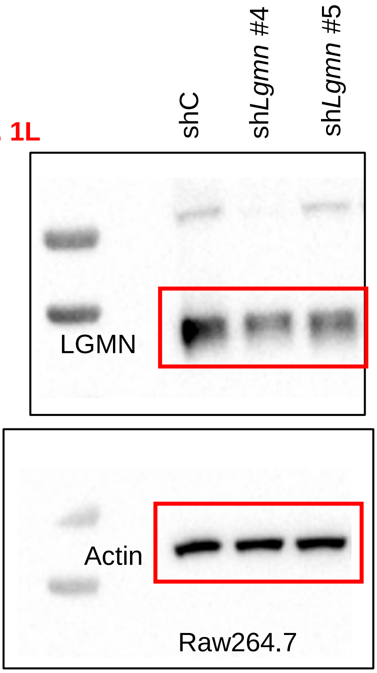

Fig. 1L

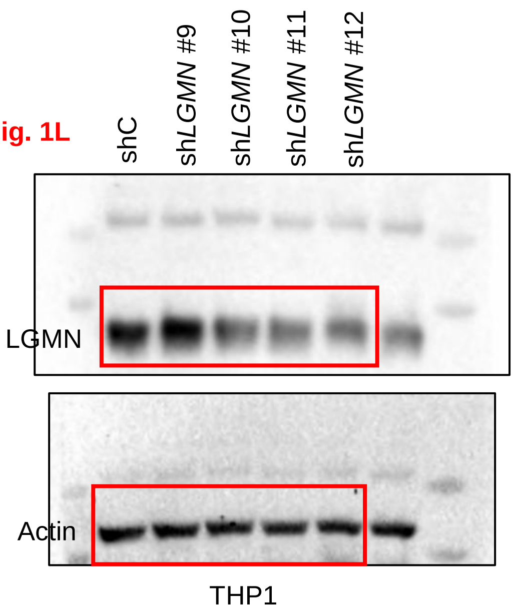

Fig. 4D

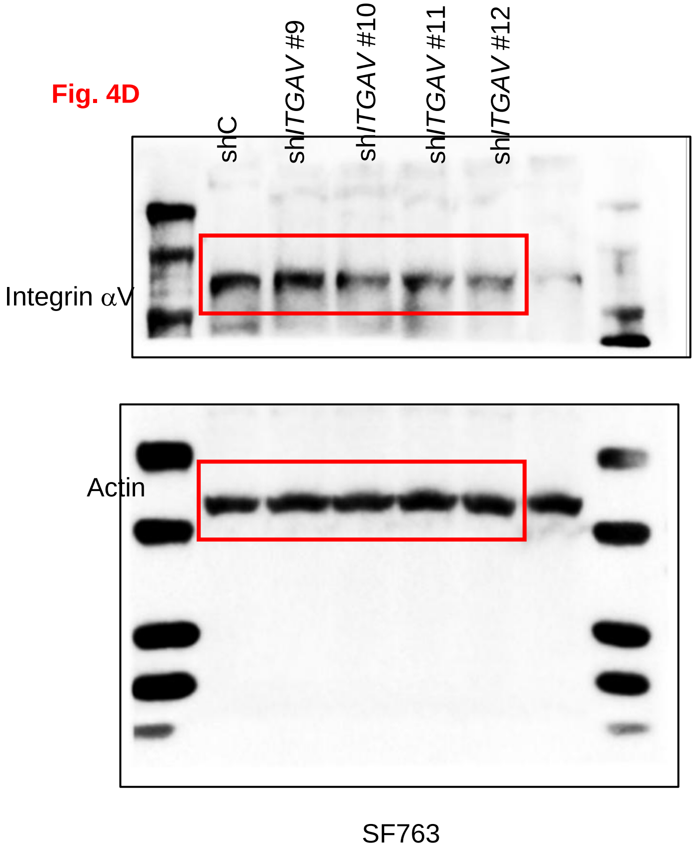

Fig. 4D

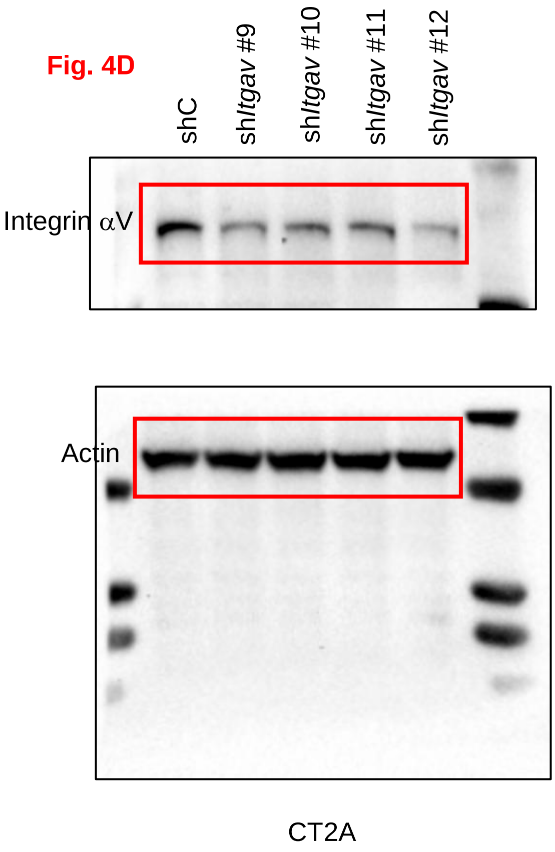

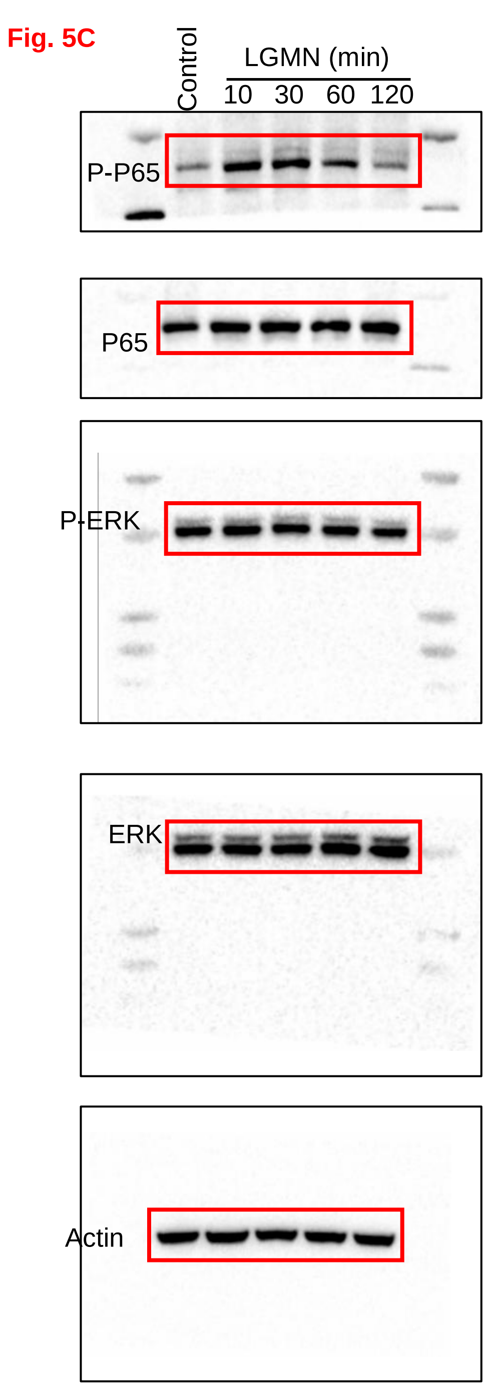
Fig. 5C
Control
LGMN (min)
10 30 60 120
P-P65
P65
P-ERK
ERK
Actin

SF763

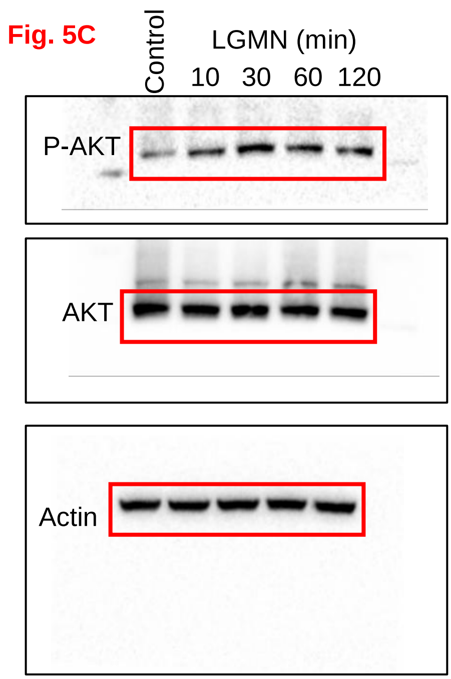
Fig. 5C
Control
LGMN (min)
10 30 60 120
P-AKT
AKT
Actin

SF763

Fig. 5D

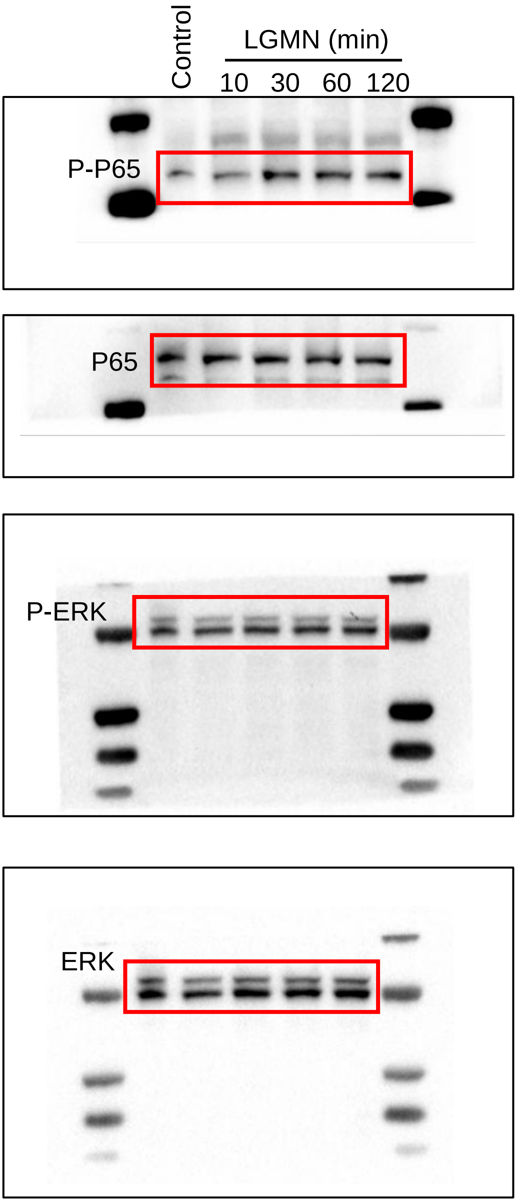

CT2A

Fig. 5D

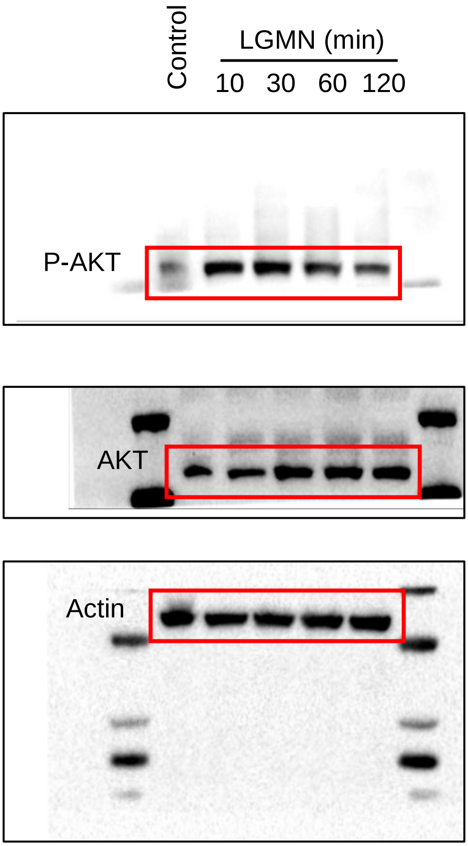

CT2A

Fig. 5E

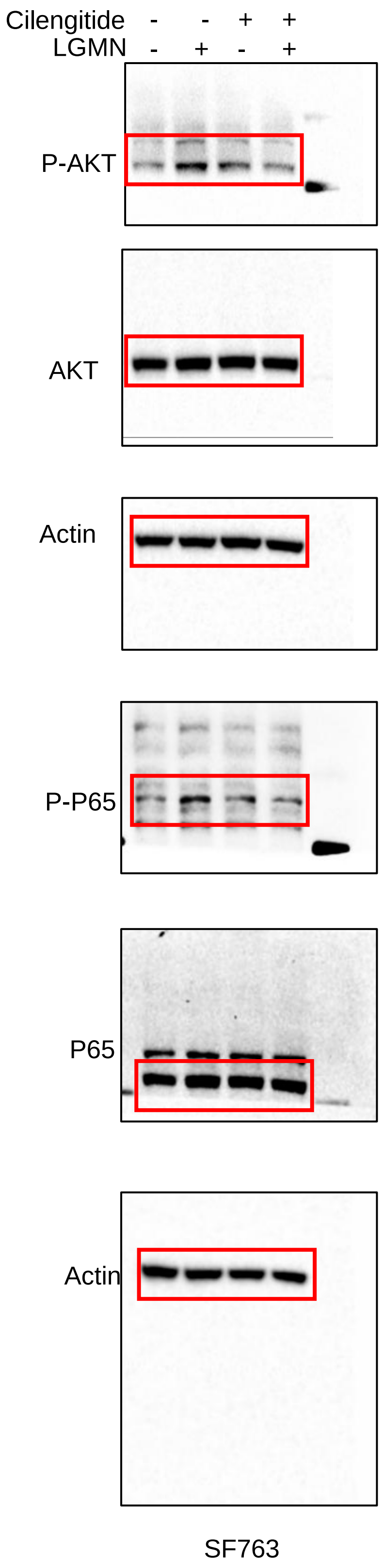
Cilengitide - - + +
LGMN - + - +
P-AKT
AKT
Actin
P-P65
P65
Actin
SF763

Fig. 5F

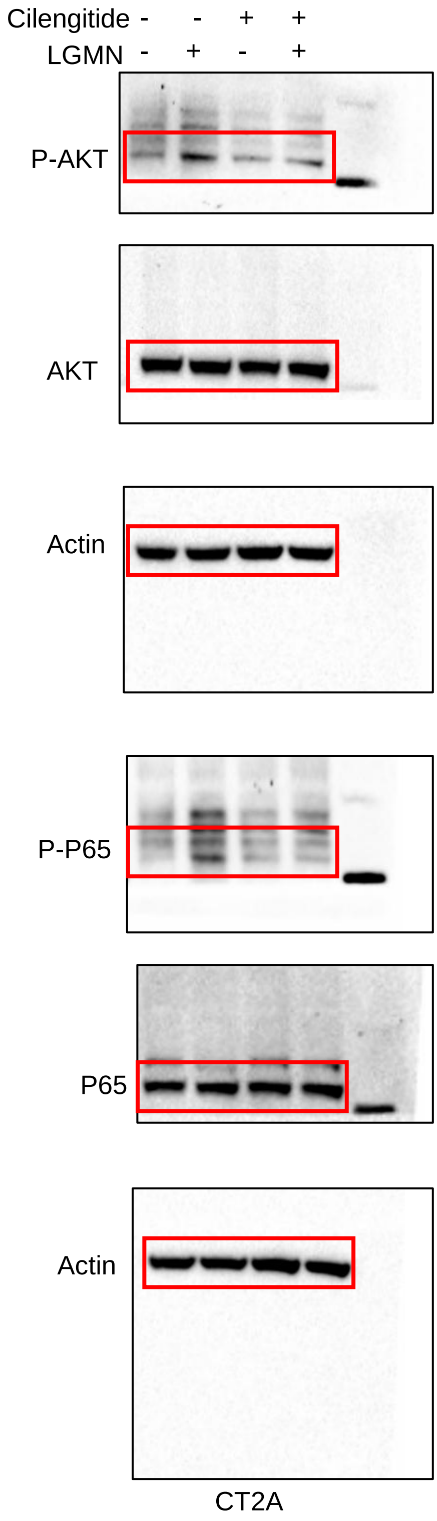
Cilengitide - - + +
LGMN - + - +
P-AKT
AKT
Actin
P-P65
P65
Actin
CT2A

Fig. 5G

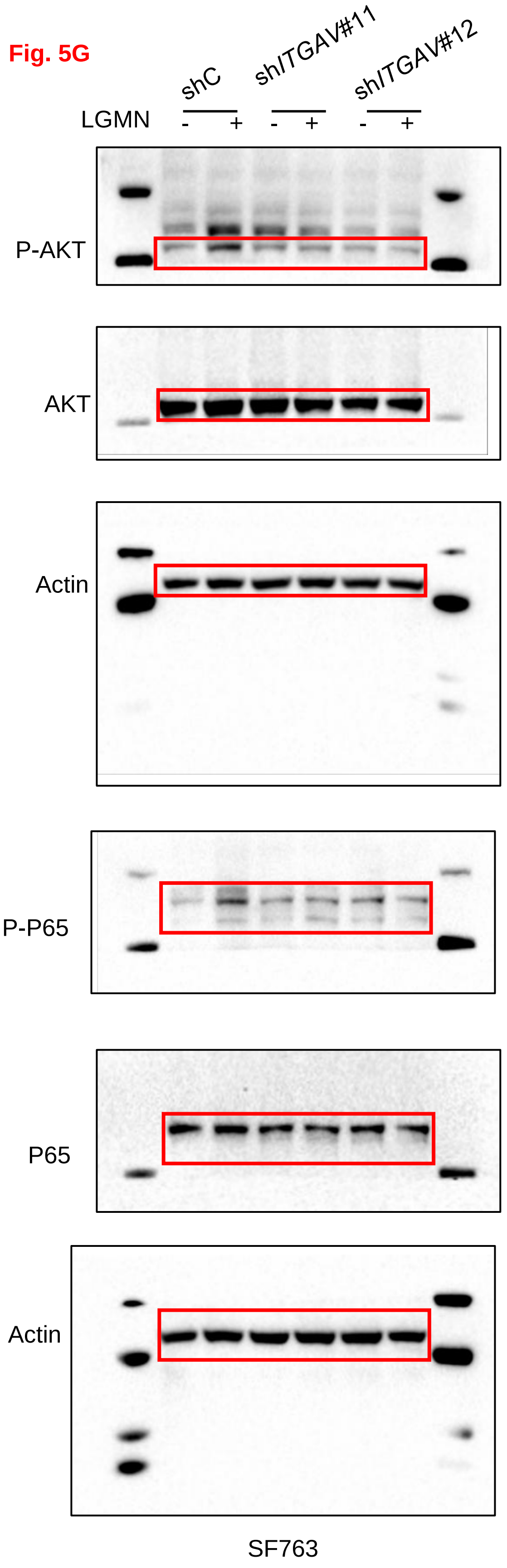
shC
sh*ITGAV*#11
sh*ITGAV*#12
LGMN
- + - + - +
P-AKT
AKT
Actin
P-P65
P65
Actin
SF763

Fig. 5G

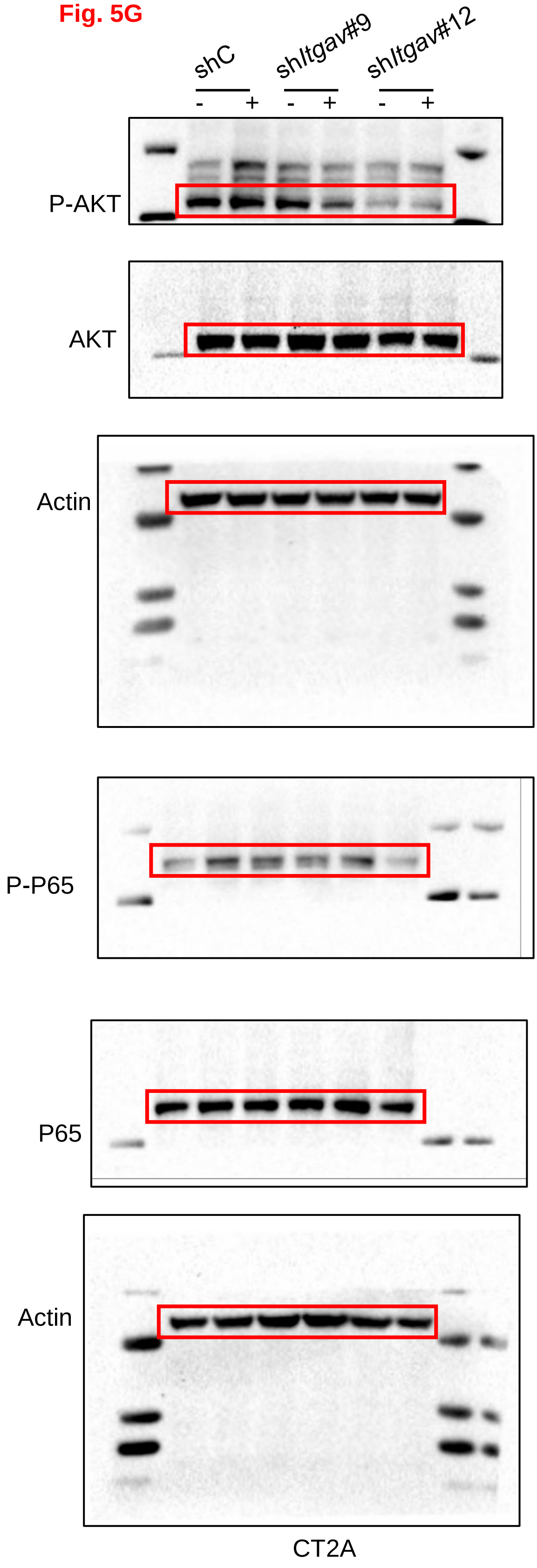
shC
sh*Itgav*#9
sh*Itgav*#12
- + - + - +
P-AKT
AKT
Actin
P-P65
P65
Actin
CT2A

Fig. 7A

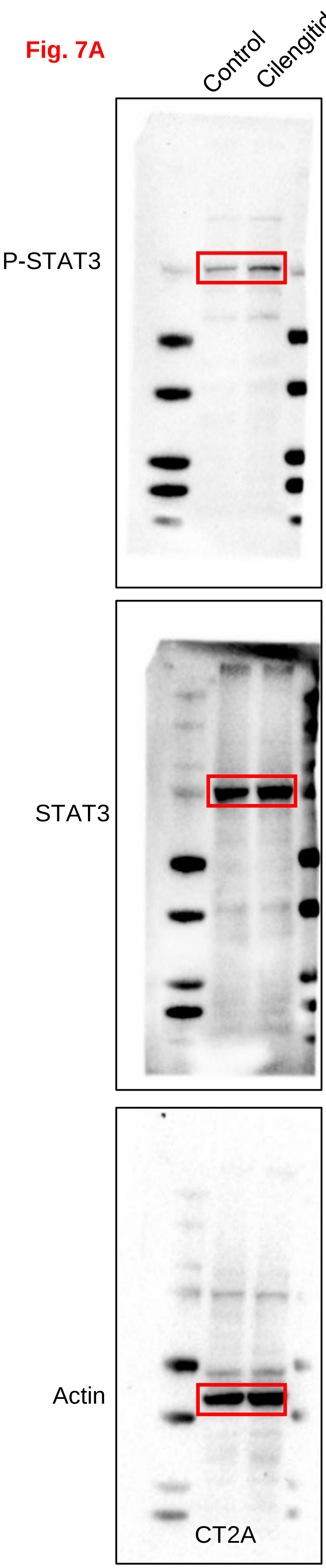
Control
Cilengitide
P-STAT3
STAT3
Actin
CT2A

Fig. 7B

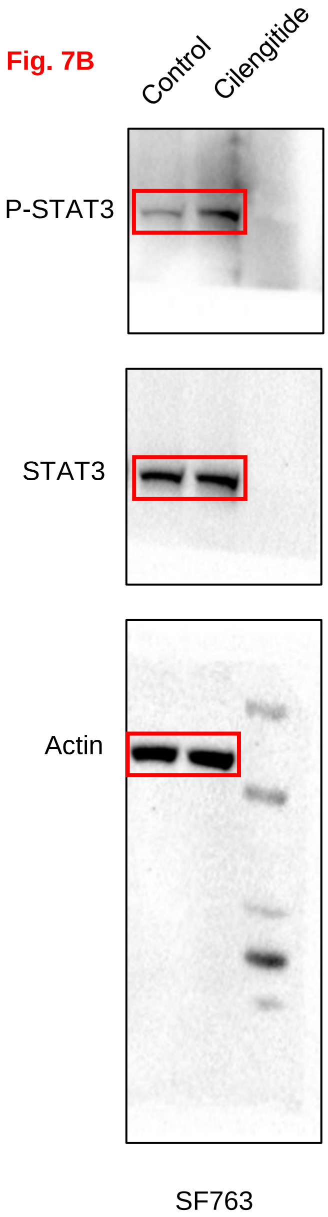
Control
Cilengitide
P-STAT3
STAT3
Actin
SF763

Fig. 7C

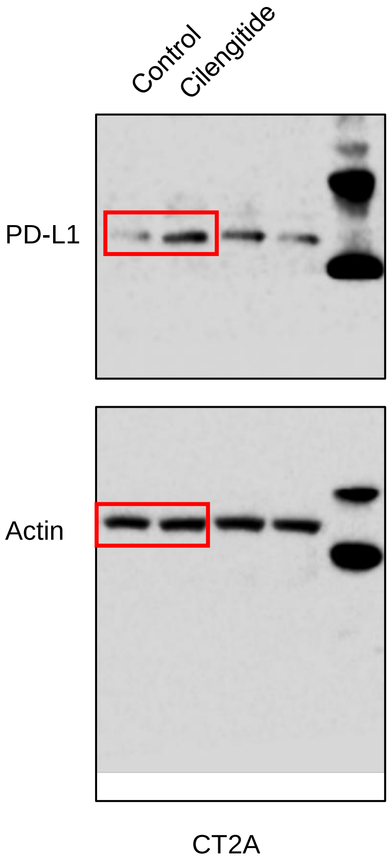

Fig. 7D

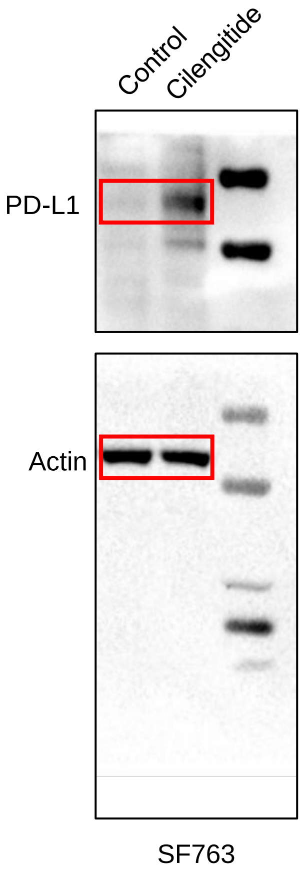

Fig. 7E

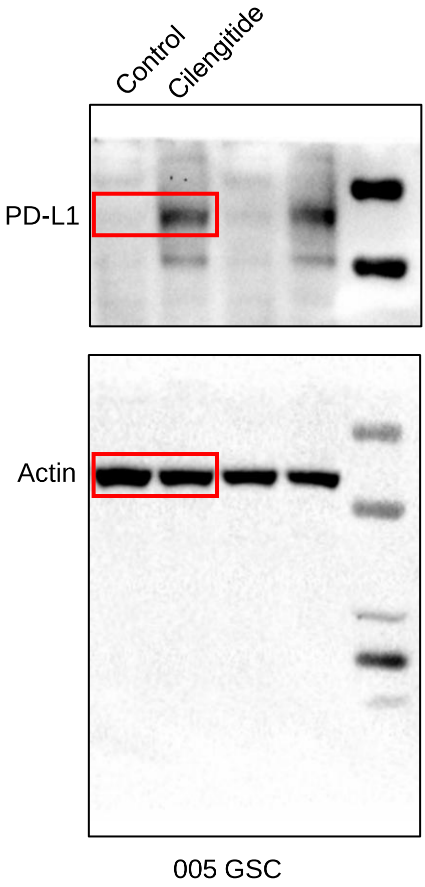

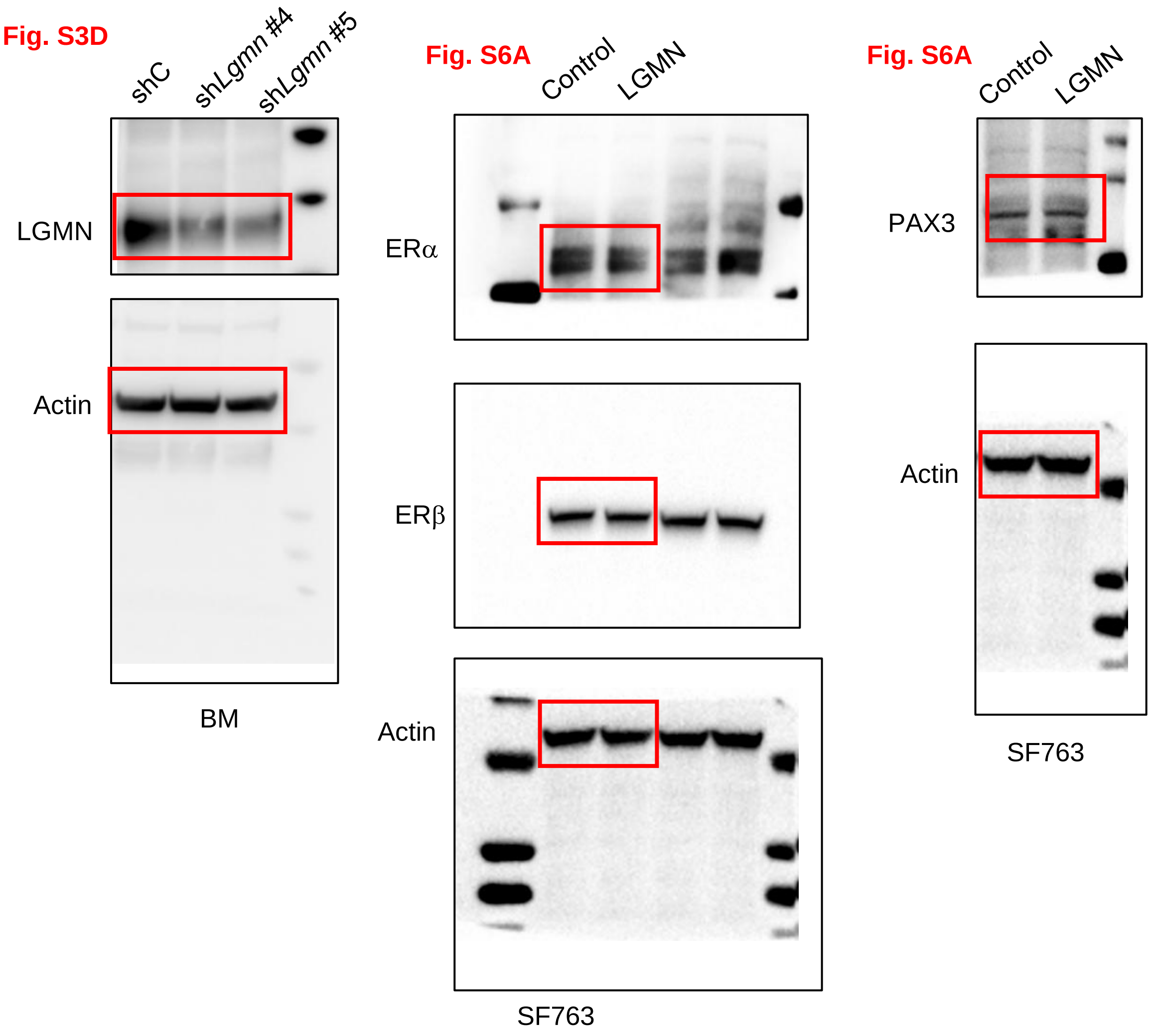

Fig. S3D
shC
shLgmn #4
shLgmn #5
LGMN
Actin
BM
Fig. S6A
Control
LGMN
ERα
ERβ
Actin
SF763
Fig. S6A
Control
LGMN
PAX3
Actin
SF763

Fig. S6C

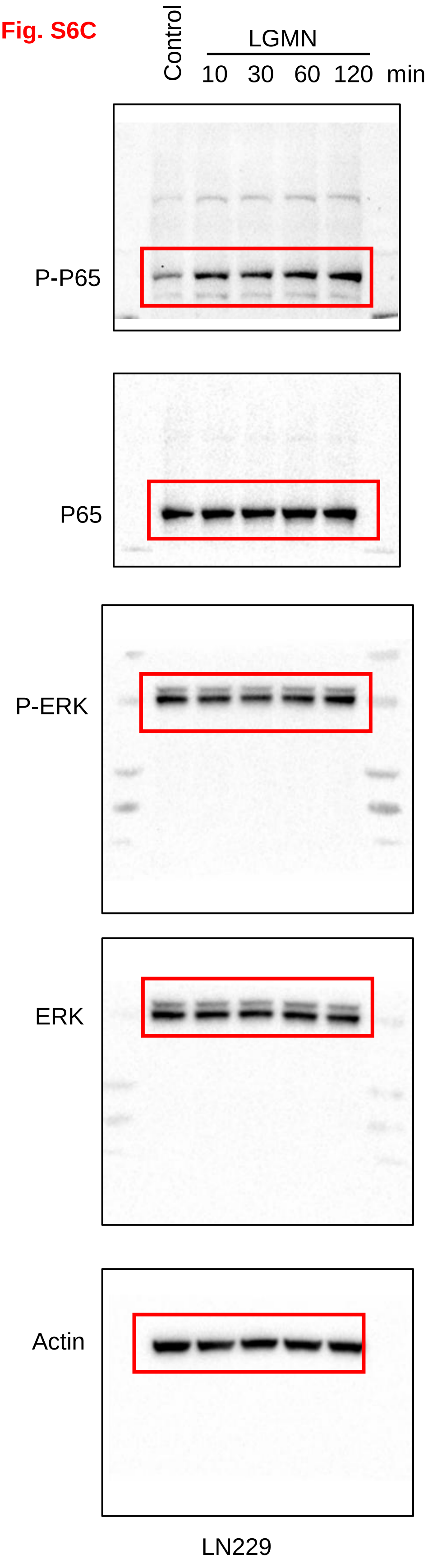

Fig. S6C

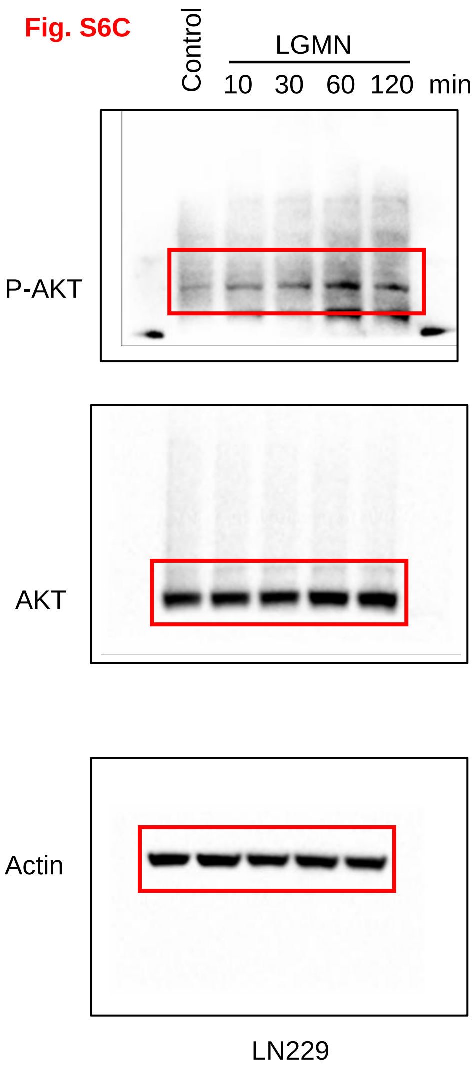

Fig. S6D

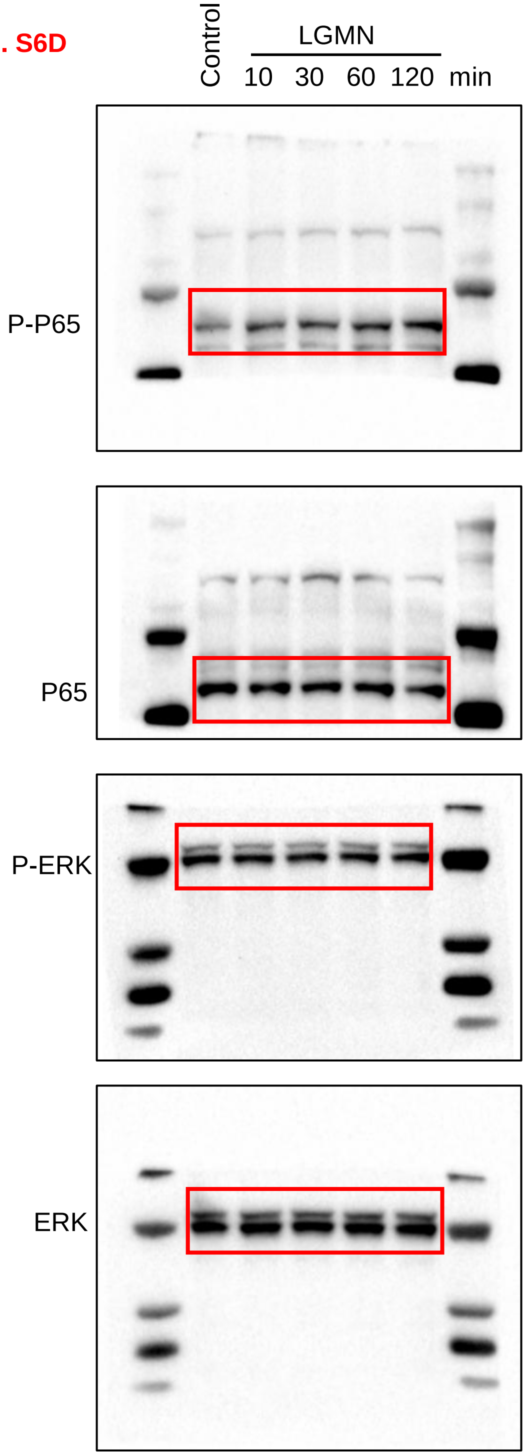

Fig. S6D

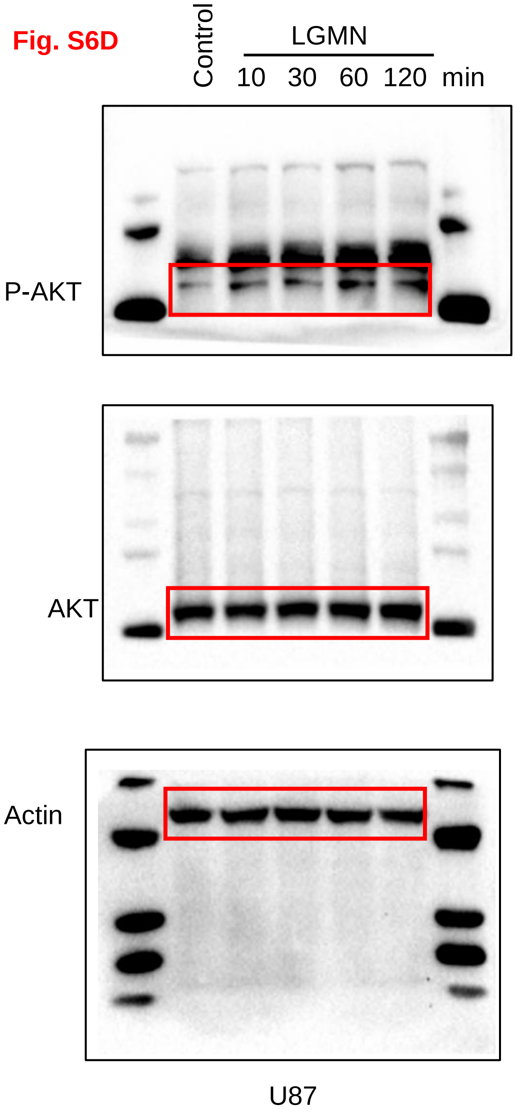

Fig. S6H

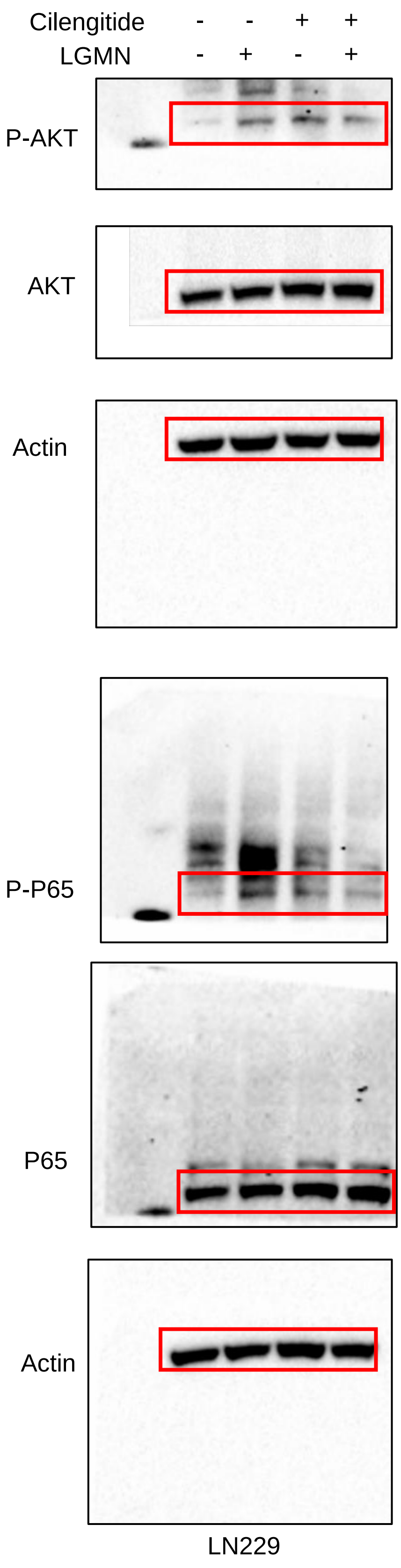

Fig. S6I

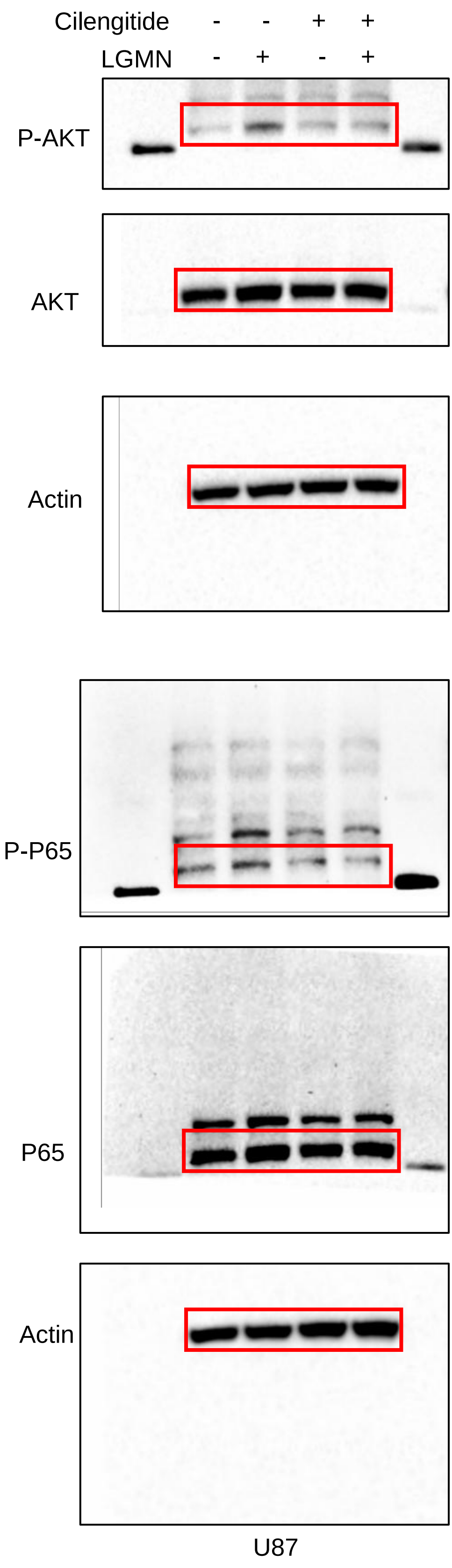

Fig. S6L

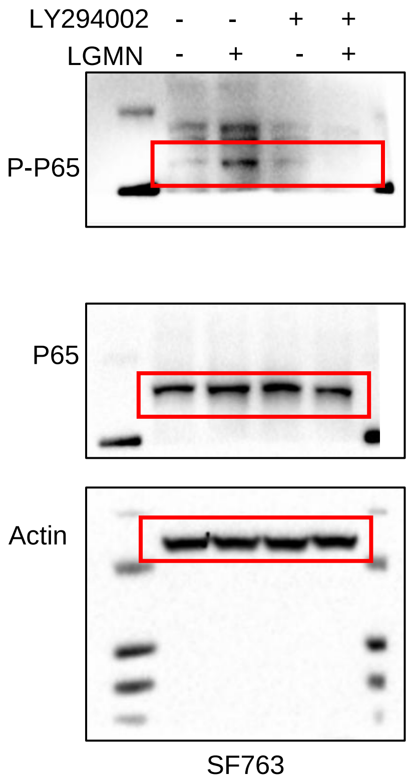

Fig. S6M

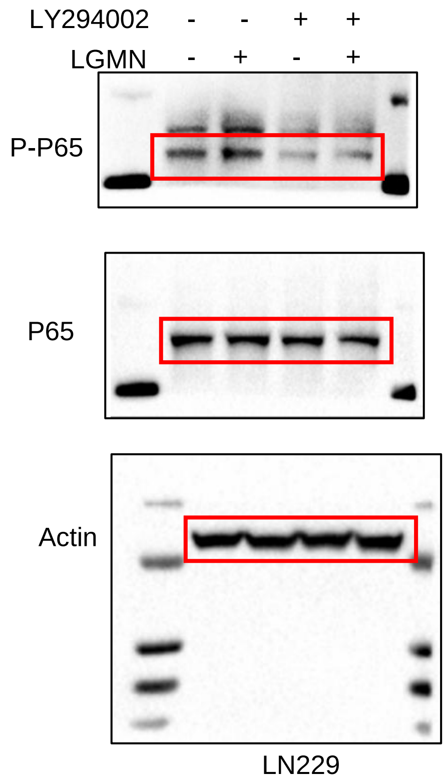

Fig. S6N

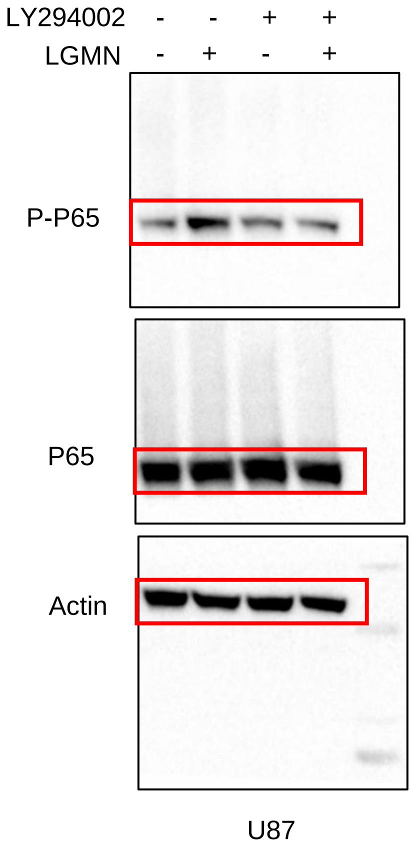

Fig. S6O

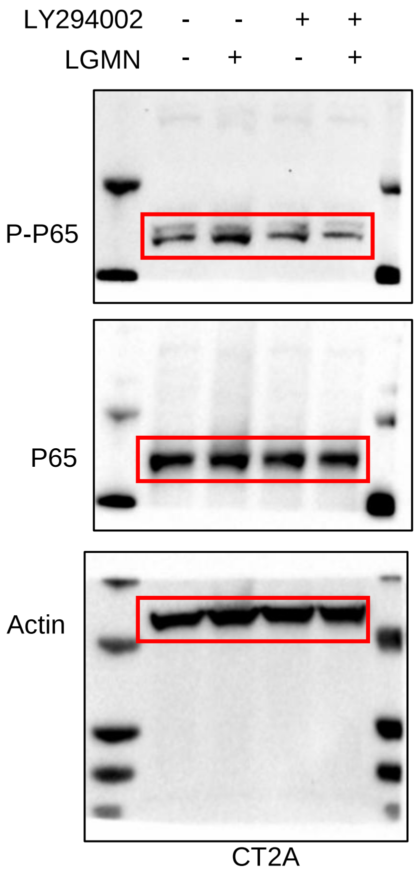

Fig. S6P

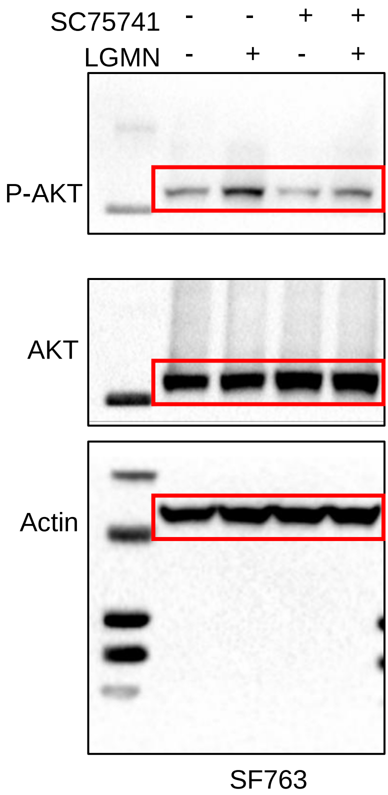

Fig. S6Q

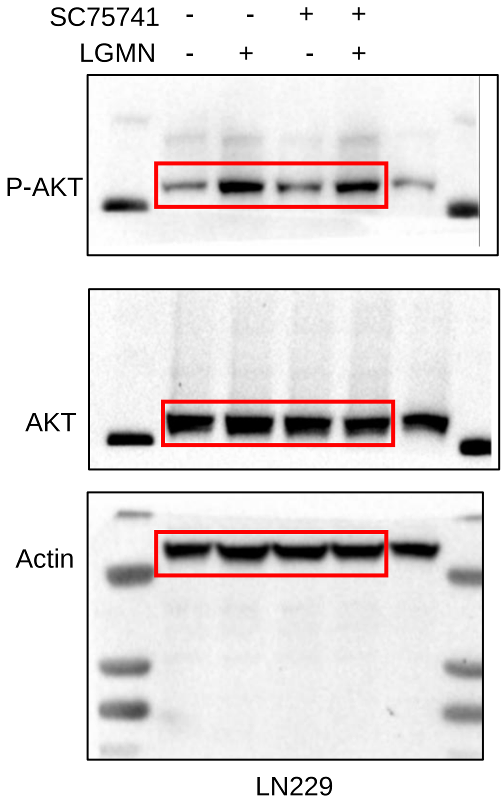

Fig. S6R

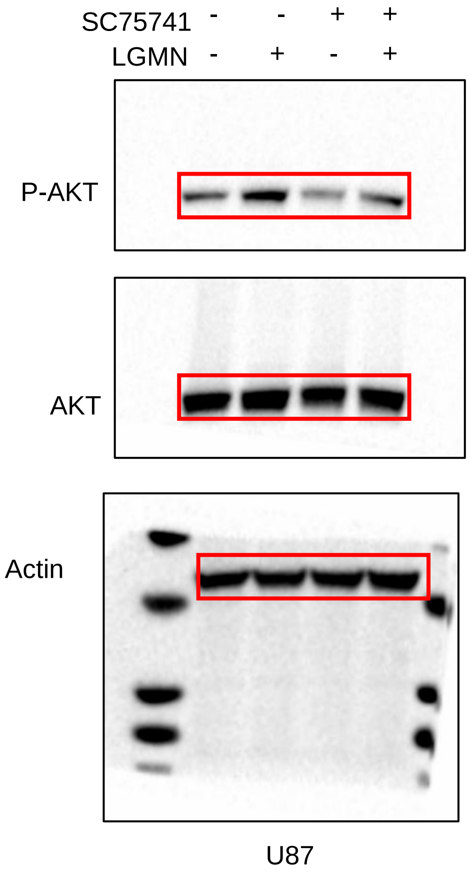

Fig. S6S

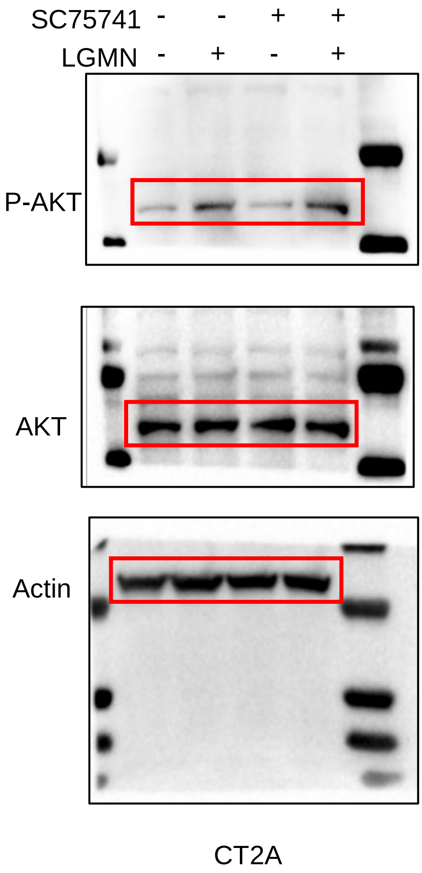

Supplement: Unedited blot and gel images [file jci-135-186034-s059.pdf]
